# Glycoside Hydrolase Family 16 Enzyme RsEG146 From *Rhizoctonia solani* AG1 IA Induces Cell Death and Triggers Defence Response in *Nicotiana tabacum*

**DOI:** 10.1111/mpp.70075

**Published:** 2025-03-17

**Authors:** Chen Chen, Dongyang Jiang, Xi Li, Xue Ji, Rui Yang, Yuwen Chen, Ying Chen, Shimin Zuo, Xijun Chen

**Affiliations:** ^1^ College of Plant Protection Yangzhou University Yangzhou Jiangsu China; ^2^ Joint International Research Laboratory of Agriculture and Agri‐Product Safety of Ministry of Education of China Yangzhou University Yangzhou Jiangsu China; ^3^ Key Laboratory of Crop Genetics and Physiology of Jiangsu Province/Key Laboratory of Plant Functional Genomics of the Ministry of Education College of Agriculture, Yangzhou University Yangzhou Jiangsu China

**Keywords:** cell death, glycoside hydrolase family 16, immune responses, *Rhizoctonia solani* AG1 IA, spray‐induced gene silencing

## Abstract

*Rhizoctonia solani* AG1 IA is a harmful necrotrophic fungus responsible for various crop diseases, including maize and rice sheath blight, which can lead to significant production losses. However, the pathogenic mechanisms and the roles of effectors in this pathogen remain poorly understood. In this study, we identified a glycoside hydrolase 16 family gene, *RsEG146*, from *R. solani* that was upregulated during its infection of *Zea mays* leaves. When transiently expressed through agroinfiltration, RsEG146 induced cell death in the leaves of tobacco ( *Nicotiana tabacum* ‘Samsun’). The predicted signal peptide of RsEG146 was essential for its cell death‐inducing activity, while the conserved enzymic active site was not required. The chitin‐binding domain was critical for the cell death‐inducing activity of RsEG146, with Gly47 identified as the key residue. Substitution of Gly47 with aspartate, glutamate, or proline significantly impaired the cell death‐inducing activity of RsEG146. Additionally, transient and heterogeneous expression of RsEG146 enhanced the pathogenicity of *Botrytis cinerea* on tobacco, and silencing this gene through spray‐induced gene silencing (SIGS) reduced the severity of the disease in maize, indicating that RsEG146 functions as an effector. Furthermore, RsEG146 triggered a plant immune response in tobacco. This study demonstrates that RsEG146 is a potential effector and plays a crucial role in the interactions between *R. solani* AG1 IA and its host.

## Graphical abstract

We identified a glycoside hydrolase 16 family member RsEG146 from *Rhizoctonia solani* AG1 IA that acts as an effector by inducing *Nicotiana tabacum* cell death via its chitin‐binding domain.

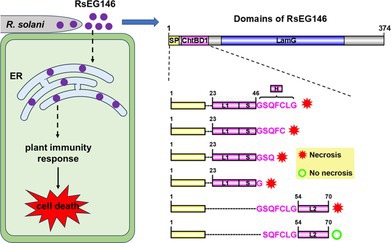

## 1 Introduction

*Rhizoctonia solani* (teleomorph *Thanatephorus cucumeris*) is a necrotrophic fungal pathogen with a broad host range that can infect numerous economically significant monocots and dicots, including rice, wheat, maize, cotton, soybean and tobacco (Chaudhary et al. 2019; Chen et al. 2019; Tziros and Karaoglanidis 2022). This pathogen does not produce conidia; instead, it generates sclerotia to survive in the soil and disseminate through water in agricultural fields (Li et al. 2019). *R. solani* is primarily classified into 14 anastomosis groups (AGs) based on hyphal fusion (AG1–AG13, AGBI), with the AG1 IA subgroup being the most virulent (Ogoshi 1987; Anderson et al. 2017; Dölfors et al. 2019). *R. solani* AG1 IA predominantly infects the leaves and sheaths of rice and maize plants, leading to reduced seed setting rates and grain weight, which results in significant yield losses in rice production in China (Bernardes‐de‐Assis et al. 2009; Chen et al. 2019; Sharma et al. 2020). During the infection of plant hosts, *R. solani* produces a variety of molecules or proteins, including toxins and cell wall‐degrading enzymes that facilitate its colonisation (Ren et al. 2017; Chen et al. 2017; Niu et al. 2023). However, fewer studies have focused on the effectors of *R. solani* compared to other proteins. Although various candidate effectors have been identified through genome and transcriptome analyses (Zheng et al. 2013; Hane et al. 2014; Wibberg et al. 2014, 2016; Anderson et al. 2017; Ghosh et al. 2018), the number of verified functional effectors remains limited (Li et al. 2019; Wei et al. 2020), and their specific functions are largely unknown.

The plant cell wall serves as a protective barrier against various environmental stresses. Among these stresses, biotic threats are significant, as pathogens secrete cell wall‐degrading enzymes (CWDEs) to compromise the integrity of the cell wall, facilitating further invasion and colonisation (Kubicek et al. 2014). CWDEs primarily consist of cellulases, pectinases, and xylanases, which specifically hydrolyse the main components of plant cell walls, including cellulose, pectin, and xylan. These enzymes are generally recognised as critical pathogenic factors (Brito et al. 2006; Lagaert et al. 2009). For example, disruption of the xylanase gene *xyn11A* in *Botrytis cinerea* decreases the level of extracellular xylanase activity and virulence (Brito et al. 2006); overexpression of the pectin lyase gene *CcpelA* in *Colletotrichum coccodes* increases fungal aggressiveness towards tomato and disruption of this gene decreases the virulence of the pathogen (Ben‐Daniel et al. 2011). CWDEs induce necrosis, elicit the plant immune response as pathogen‐associated molecular patterns (PAMPs) directly, or generate damage‐associated molecular patterns (DAMPs) during the infection period. For instance, an ethylene‐inducing xylanase EIX from *Trichoderma reesei* elicits plant defence responses in tobacco ( *Nicotiana tabacum* ) and tomato (Ron and Avni 2004); endoglucanases VdEG1, VdEG3, and pectate lyase VdPEL1 secreted from *Verticillium dahliae* induce the plant immune response of *Nicotiana benthamiana* and cotton (Gui et al. 2017; Yang et al. 2018a); glucanase CfGH17‐1 from *Cladosporium fulvum* breaks the cell wall and releases a DAMP to induce tomato cell death (Ökmen et al. 2019); xyloglucanase BcXYG1 and xylanase BcXyl1 from *B. cinerea* , and XEG1 from *Phytophthora sojae* also trigger the plant immunity response (Ma et al. 2015b; Zhu et al. 2017; Yang et al. 2018b); the secreted endoglucanases MoCel12A and MoCel12B from *Magnaporthe oryzae* induce the immunity response of rice by releasing oligosaccharides as DAMPs from the host cell wall (Yang et al. 2021).

The genome of *R. solani* contains large numbers of CWDE‐coding genes belonging to different glycoside hydrolase (GH) families, which have been widely reported as important pathogenic factors (Li et al. 2021; Rafiei et al. 2021). A number of the GH28 family, polygalacturonase (PGs), secreted by *R. solani* , cause damage to cell membranes and maceration of rice leaves and sheaths (Chen et al. 2017). Inhibition of PG enzymatic activity leads to the accumulation of oligogalacturonides (OGs) (De Lorenzo and Ferrari 2002). Subsequently, wall‐associated kinases (WAKs) recognise these OGs, enhancing host resistance by activating defence responses (Ferrari et al. 2013; Gramegna et al. 2016). The typical CWDE PAMPs of *R. solani* , specifically the GH45 member endo‐glucanohydrolase EG1, induce plant defence responses, including the expression of defence genes, cell death, and a burst of reactive oxygen species (ROS) (Ma et al. 2015). Additionally, the PAMP sites of EG1 have also been identified (Guo et al. 2021). However, the specific roles of most CWDEs in *R. solani* virulence remain largely unknown.

Plants and pathogens have evolved elaborate interaction systems against each other (Zipfel 2008; Zhai et al. 2021). Pattern recognition receptors (PRRs) located on the surfaces of plant cells recognise conserved PAMPs or DAMPs derived from host plants. This recognition induces the first layer of plant innate immunity, known as PAMP‐triggered immunity (PTI), which aims to prevent pathogens from further colonisation and invasion (Couto and Zipfel 2016; Bacete et al. 2018). Conversely, pathogens secrete numerous effectors to disrupt PTI and promote infection, called effector‐triggered susceptibility (ETS) (Jones and Dangl 2006; Saijo et al. 2017; Anderson et al. 2017). In response, host plants produce resistance (R) proteins to recognise these effectors, resulting in a more robust immune response termed effector‐triggered immunity (ETI), which is associated with heightened immune responses, such as the hypersensitive response (HR) (Houterman et al. 2008; Stergiopoulos and de Wit 2009; Lolle et al. 2020). Effectors are less‐conserved molecules, secreted or translocated from pathogens, that have no obvious effect on the growth and development of the pathogens but induce the host defence response or promote pathogen infection, so effectors are essential in pathogen–host interaction (Todd et al. 2022). Effectors studied in *R. solani* include RSAG8_06778, RsLysM, AGLIP1, RsRlpA, RsIA_NP8, RsSCR10, RsCRP1 and AOS2, RsMf8HN and RsIA_CtaG/Cox11 (Anderson et al. 2017; Dölfors et al. 2019; Li et al. 2019; Charova et al. 2020; Tzelepis et al. 2021; Wei et al. 2020; Niu et al. 2021; Niu et al. 2023; Yang et al. 2023; Zhang et al. 2024). Among these effectors, AGLIP1, RsIA_NP8, RsSCR10, and RsIA_CtaG/Cox11are secreted by *R. solani* AG1 IA, and they all trigger plant cell death and induce host immune response (Li et al. 2019; Wei et al. 2020; Niu et al. 2021; Zhang et al. 2024).

The GH16 family is a polyspecific group of enzymes capable of hydrolysing and/or glycosylating β‐glucans and β‐galactans (Lombard et al. 2014). Members of this family play crucial roles in the degradation and remodelling of plant and fungal cell walls, the degradation of fungicides, and biomass degradation (Meng et al. 2016; McGregor et al. 2017; Fang et al. 2019; Viborg et al. 2019). They also function as effectors and elicitors of plant defence responses. A GH16 family member, BcCrh1 from *B. cinerea* , which contains a single conserved domain, is essential for fungal development and induces plant cell death and defence responses. Notably, the cell death‐inducing activity of BcCrh1 is unrelated to its plant cell wall‐degrading activity (Bi et al. 2021). In this study, we discovered that the GH16 member gene *RsEG146*, derived from *R. solani* AG1 IA, is upregulated during infection, leading to tobacco leaf necrosis and host defence responses. Furthermore, a specific amino acid residue of RsEG146 is sufficient to elicit cell death. Thus, RsEG146 is a potential effector and is crucial in *R. solani* AG1 IA–host interactions. Our findings provide essential insights into the molecular mechanisms underlying host– *R. solani* interactions and identify a potential target gene for the development of fungicides to control crop diseases caused by *R. solani* .

## 2 Results

### 2.1 Cloning, Sequence Analysis and Expression of *RsEG146*

**FIGURE 1:**
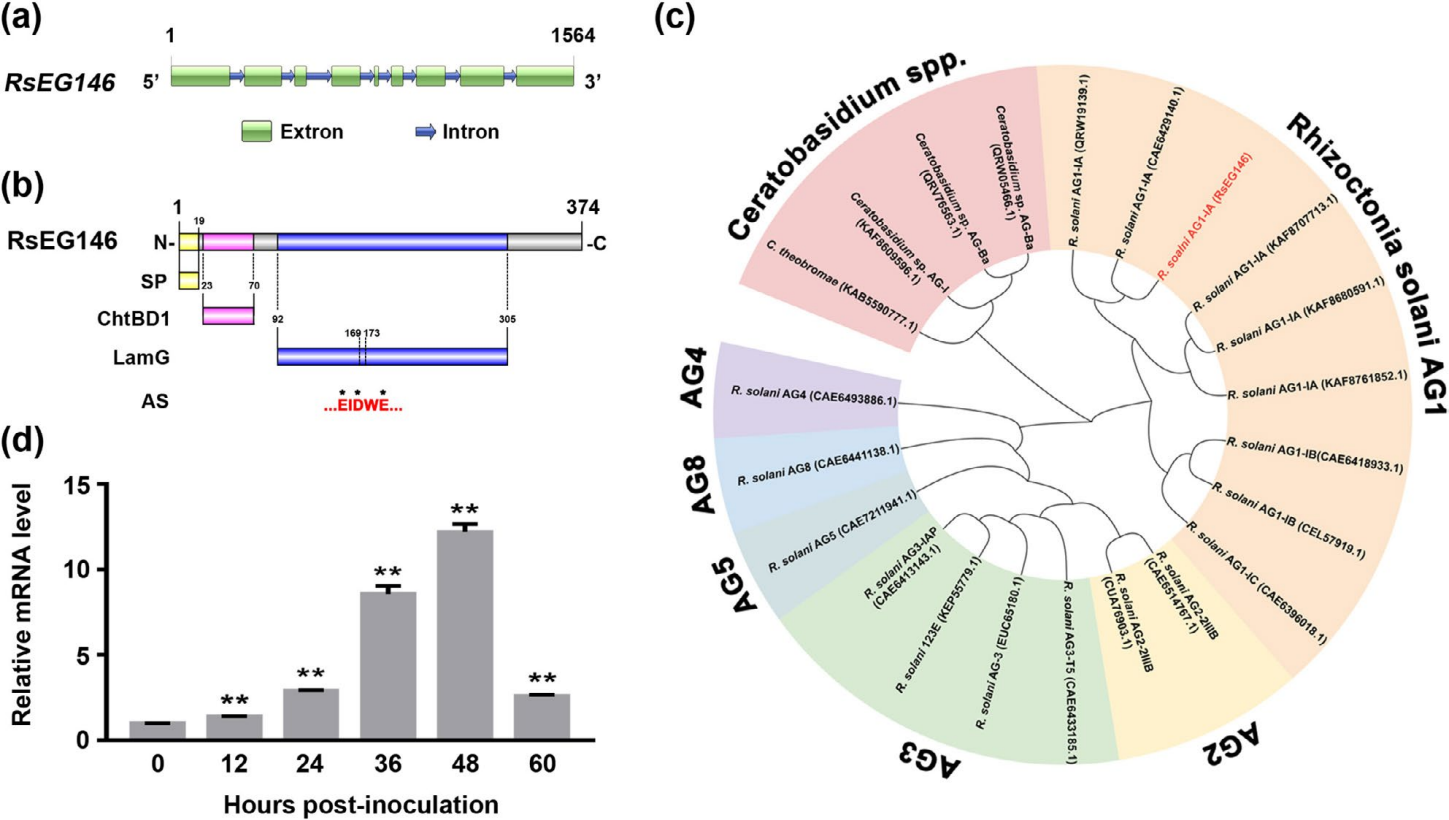
Sequence analysis and expression characters of *RsEG146*. (a) Total nucleotide sequence of *RsEG146* amplified from *Rhizoctonia solani* AG1‐IA isolate YN‐7. The full‐length sequence of *RsEG146* contains 1564 bp including nine exons and eight introns. (b) Conserved domain analysis of RsEG146 by using Conserved Domain Database. SP, signal peptide; ChtBD1, type 1 chitin‐binding domain; Lam G, laminin G domain; AS, active site. (c) Cladogram of glycoside hydrolase family 16 members in different anastomosis groups of *R. solani* and *Ceratobasidium* by using similar amino acid sequences of RsEG146. (d) Maize leaves inoculated with *R. solani* AG1 IA were collected 0, 12, 24, 36, 48 and 60 h post‐inoculation for detecting gene expression level by reverse transcription‐quantitative PCR. *RsGAPDH* expression was used as an internal reference gene. Column = mean ± SE (**p < 0.01).

The total sequence and open reading frame (ORF) of *RsEG146* were amplified from genomic DNA and cDNA of *R. solani* AG 1‐IA isolate YN‐7. The full‐length sequence of *RsEG146* contains 1565 bp, including nine exons and eight introns (Figure 1a). The CDS of RsEG146 is 1125 bp, encoding 374 amino acid residues with a termination codon. A signal peptide (SP) was predicted at amino acids (aa) 1–19 of RsEG146 using SignalP (Figure 1b). A BLAST search for *RsEG146* revealed that it is located on chromosome 4, and the nucleotide sequence shares 99.71% identity with a glycoside hydrolase family 16 protein gene (GenBank number: XM_043320364.1) in the *R. solani* genome reported by Li et al. (2021). Phylogenetic analysis showed that homologous amino acid sequences of RsEG146 are widespread in Basidiomycota fungi (Figure S1), and they are clearly distinguished between *Ceratobasidium* spp. and each *R. solani* AG (Figure 1c).

There are 20 GH16 coding genes in the genome of *R. solani* AG1 IA, which can be separated into two superfamily groups and an unpredicted domain based on their amino acid sequences (Figure S2), of which the LamG superfamily is divided into two clusters with different active sites, in accord with studies by Viborg et al. (2019). Conserved domain analysis of RsEG146 suggests that it possesses two conserved domains, the type 1 chitin‐binding domain (ChtBD1) and the laminin G‐like domain (LamG), a unique domain combination in GH16 of *R. solani* (Figure 1b; Figure S3). The active site (AS) was predicted in LamG with the motif of EIDWE (169–173 aa) that is highly conserved in most GH16 family enzymes (Figure 1b) (Viborg et al. 2019). The modelled ChtBD1 domain has two anti‐parallel β‐sheets followed by a short α‐helix, including one disulphide bond. The LamG structure model showed a convex and a concave face that consists of a GH16 family β‐jelly‐roll fold stacked by six‐stranded and seven‐stranded β‐sheets. The predicted active site is also in the β‐jelly‐roll fold as other GH16 family enzymes (Figure S3).

To investigate the expression profile of RsEG146 during *R. solani* AG1 IA infection, we studied *RsEG146* expression on *Zea mays* leaves. The expression levels of *RsEG146* increased from 0 to 48 h after injection and reached 10‐fold induction at 48 h compared with 0 h (Figure 1d). These findings indicated that RsEG146 might be crucial in *R. solani* AG1 IA infection.

### 2.2 *RsEG146* Induces Cell Death and Localises at the Endoplasmic Reticulum on *N. tabacum* Leaves

**FIGURE 2:**
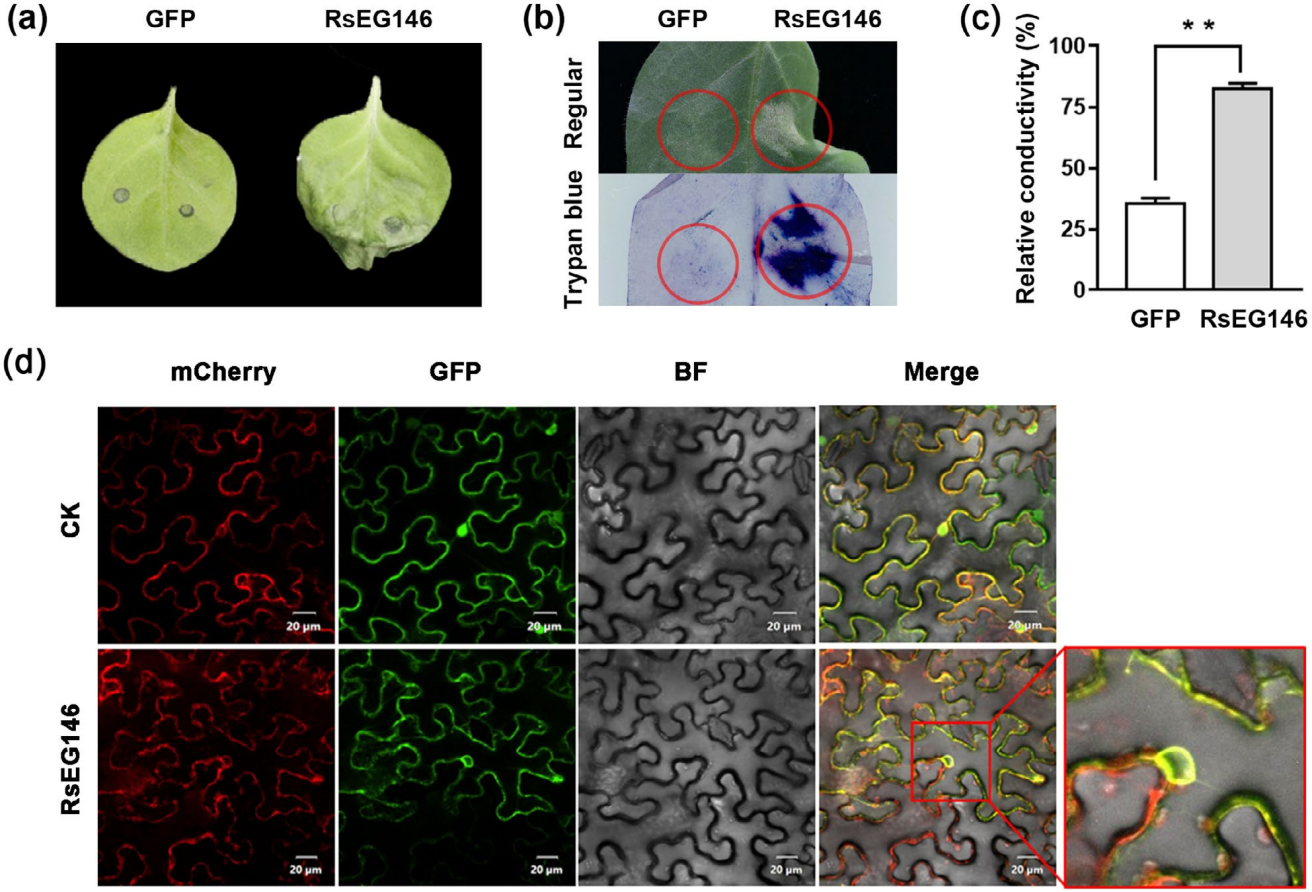
Transiently expressed *RsEG146* induced cell death and its subcelluar localisation in *Nicotiana tabacum* leaves. (a) Symptoms following transient expression of *RsEG146* in *N. tabacum* at 36 and 48 h. (b) Trypan blue staining of *N. tabacum* leaves. (c) Conductivity of *N. tabacum* leaves. Transiently expressed *GFP* was used as a negative control. (d) Subcellular localisation of RsEG146 in *N. tabacum* leaves with endoplasmic reticulum localisation signal mCherry‐HDEL. The vector pMD1‐35S‐GFP carrying GFP was used as a control. Bar = 20 μm.

Many fungal glycoside hydrolases, including GH16, were manifested to induce plant cell death (Bi et al. 2021). To identify whether RsEG146 can cause cell death, *RsEG146* was transiently expressed in *N. tabacum* leaves via *Agrobacterium tumefaciens* ‐mediated transfection. Cell death in *N. tabacum* leaves was observed at 48 h post‐inoculation (hpi), while a negative control of green fluorescent protein (GFP) did not induce cell death (Figure 2a). The necrotic part of *N. tabacum* leaves was stained by trypan blue (Figure 2b), and a remarkable increase in relative electrical conductivity was also observed in *RsEG146*‐expressing *N. tabacum* leaves (Figure 2c), which indicates that RsEG146 can induce cell death in tobacco leaves.

To investigate the subcellular localisation of RsEG146, RsEG146‐GFP fusion protein and GFP were transiently co‐expressed with endoplasmic reticulum (ER) marker mCherry‐HDEL in tobacco leaves. The fluorescence was observed under laser scanning confocal microscopy at 2 days post‐inoculation (dpi). Green fluorescence from RsEG146‐GFP and red fluorescence from mCherry‐HDEL overlapped, suggesting that RsEG146 could be recognized by receptors located in the ER to trigger cell death (Figure 2d).

### 2.3 Function Validation of the Predicted SP of RsEG146

**FIGURE 3:**
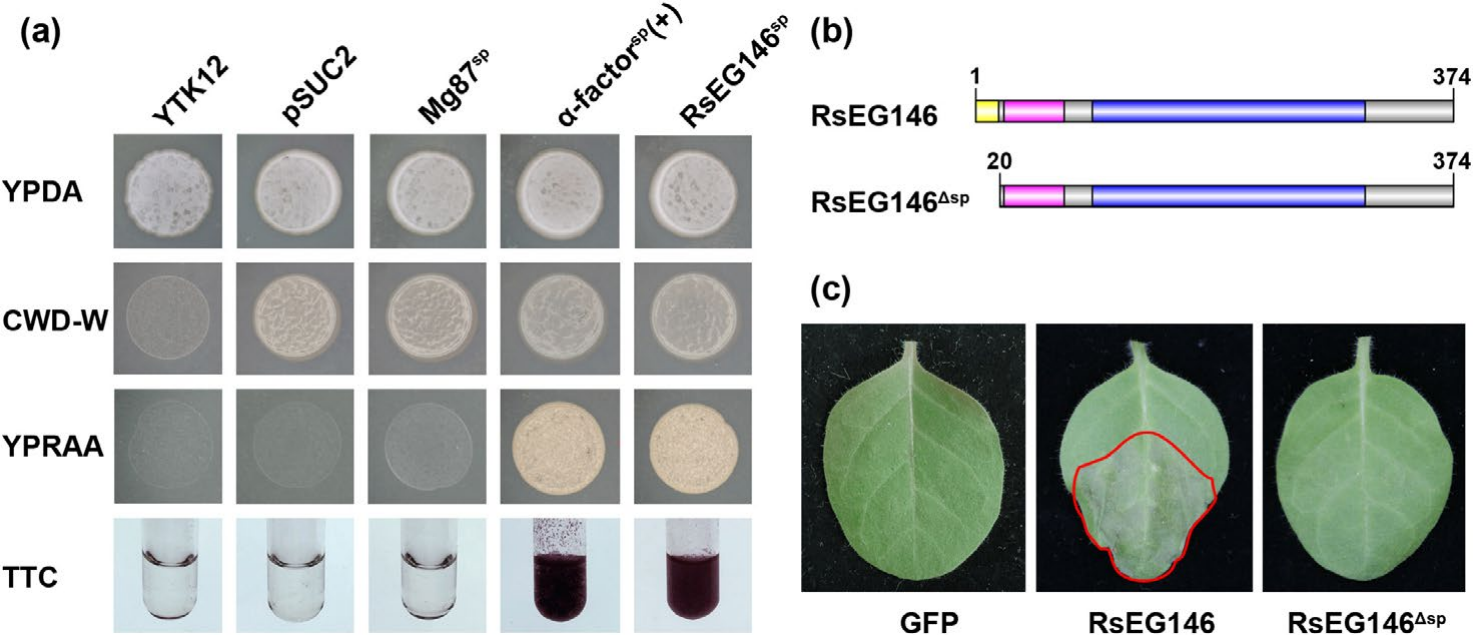
The signal peptide (SP) of RsEG146 is functional. (a) Functional validation of the SP of RsEG146 using yeast invertase secretion assay. All transformed YTK12 yeast strains grew on YPRAA medium with raffinose as the sole carbon source. α‐factor signal peptide from pPIC9K and N‐terminal sequence of Mg87 from *Magnaporthe oryzae* were used as positive and negative controls, respectively. The untransformed YTK12 did not grow on either CMD−W or YPRAA media, and the TTC solution remained colourless. Yeast growth on CMD−W medium was equally viable among the transformed strains. Mg87^SP^: Negative control SP of Mg87 from *M. oryzae* ; α‐factor^SP^: Positive control SP of α‐factor; RsEG146^SP^: SPs of RsEG146. (b) Schematic presentation of RsEG146 and RsEG146^ΔSP^. RsEG146^ΔSP^: RsEG146 without SP. (c) Functional validation of the SP of RsEG146 by deleting the SP of RsEG146. The transient expression of RsEG146^ΔSP^ in *Nicotiana tabacum* maintained the leaf in a normal state without necrosis.

To clarify the function of the predicted SP of RsEG146, a yeast secretion assay was performed as described by Lee and Rose (2012). In this assay, YTK12 strains containing RsEG146^SP^ or the secretion signal of α‐factor amplified from the eukaryotic expression vector pPIC9K (positive control) could grow on the YPRAA medium (containing raffinose), as well as convert the dye 2,3,5‐triphenyltetrazolium chloride (TTC) to the red insoluble form. In contrast, the yeast strain had a non‐functional N‐terminus of Mg87 from *Magnaporthe grisea* (negative control) that did not grow on YPRAA. The culture filtrate‐treated TTC solution also remained colourless (Figure 3a).

Many pathogenic fungi effectors require SPs to induce plant cell death (Saitoh et al. 2012; Yang et al. 2018b; Wei et al. 2020). To identify the necrosis function of RsEG146^SP^, an SP‐lacking protein, RsEG146^ΔSP^ (Figure 3b) was transiently expressed in *N. tabacum* leaves. Unlike the wild type, RsEG146^ΔSP^ did not induce necrosis of tobacco leaves and had a different subcellular localisation (Figure 3c; Figure S4). Transient expression of RsEG146^SP^ (RsEG146^N19^) in *N. tabacum* leaves also did not induce necrosis (Figure S7). These results indicate that the predicted SP of RsEG146 secretes the functional protein and does not cause necrosis independently.

### 2.4 RsEG146 Cell Death‐Inducing Activity Is Independent of Its Enzymic Activity

**FIGURE 4:**
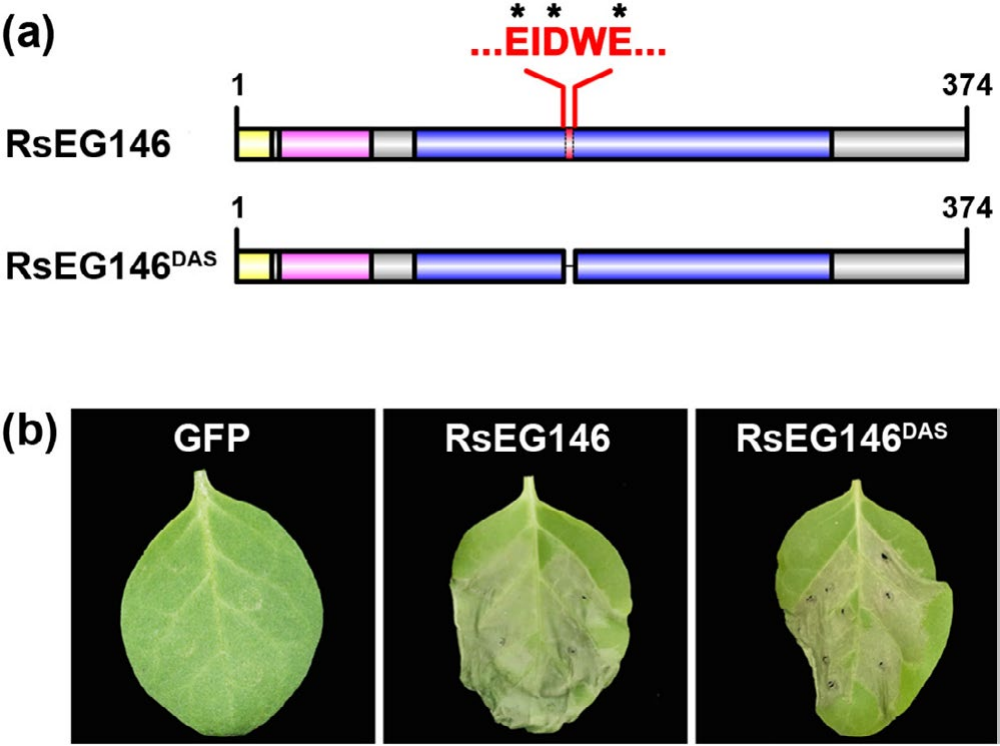
Cell death‐inducing activity of RsEG146 is independent of its enzymic activity. (a) Schematic presentation of RsEG146 and RsEG146^DAS^. RsEG146^DAS^: RsEG146 mutant with deleted active sites (DAS); EIDWE: Motif of RsEG146 enzymic active sites. *Indicates conserved amino acid residues. (b) The demonstration of relationships between enzymic activity and cell death‐inducing activity of RsEG146 by deleting enzymic active sites. The transient expression of RsEG146^DAS^ in *Nicotiana tabacum* also caused leaf necrosis.

Plant cell death caused by glycoside hydrolases may (Ben‐Daniel et al. 2011; Chen et al. 2017) or may not depend on the enzymic activity (Ma et al. 2015a; Bi et al. 2021). Prokaryotic expression of RsEG146 showed no cell wall‐degrading enzyme activity (Figure S5), while the eukaryotic expression product had little enzymic activity (Figure S6). To verify whether the glycoside hydrolase enzymatic activity of RsEG146 is required for cell death‐inducing activity, we deleted the putative active sites of RsEG146 (RsEG146^DAS^) (Figure 4a). *Agrobacterium*‐mediated transient expression of RsEG146^DAS^ on *N. tabacum* leaves also produced necrosis that was similar to the symptoms caused by wild‐type RsEG146 (Figure 4b), indicating that plant cell death‐inducing activity is independent of the enzymatic activity of RsEG146.

### 2.5 Gly47 in Chitin‐Binding Domain of RsEG146 Is Sufficient for Cell Death Elicitation

**FIGURE 5:**
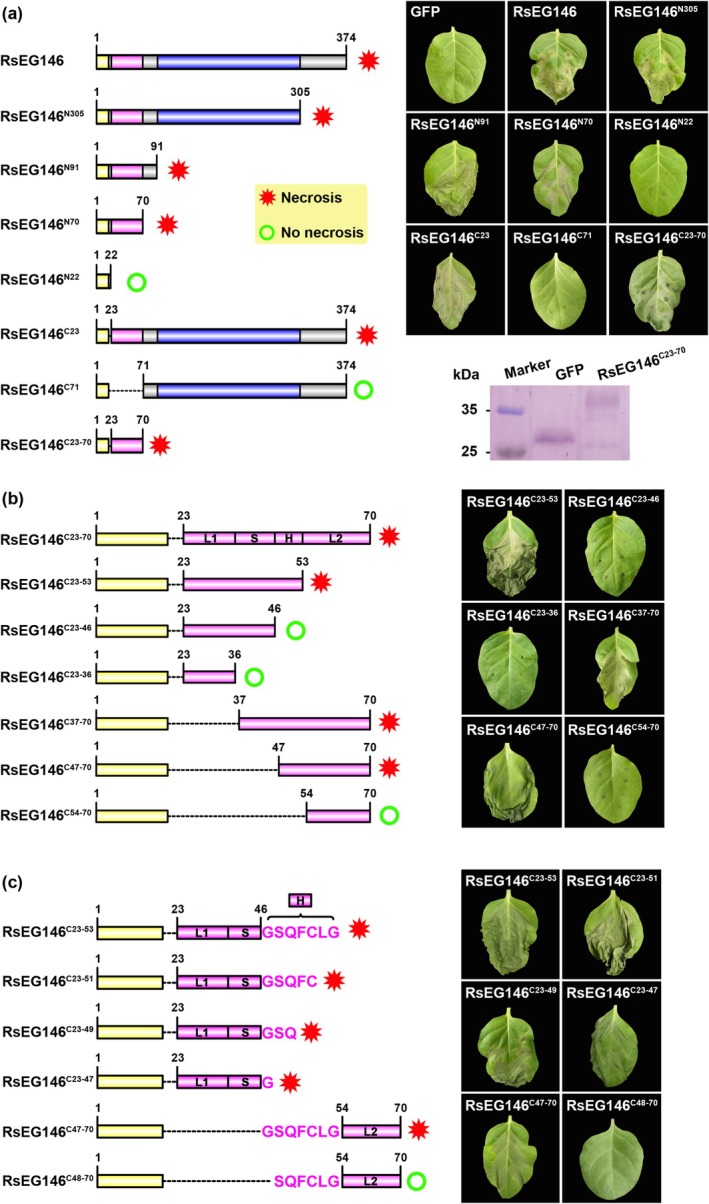
Gly47 in chitin‐binding domain of RsEG146 is sufficient for inducing cell death in *Nicotiana tabacum* . (a) Schematic presentation and corresponding leaves of different RsEG146 domains that were used in agroinfiltration assays. Western blot analysis of RsEG146^C23‐70^ production in tobacco. (b) Schematic presentation and corresponding leaves of different secondary structures of RsEG146^C23‐70^ that were used in agroinfiltration assays. (c) Schematic presentation and corresponding leaves of different amino acids in RsEG146^C47‐53^ that were used in agroinfiltration assays. All the experiments were replicated five times.

To identify the critical domain of RsEG146 for cell death induction, putative SP, ChtBD1 and LamG of RsEG146 were first analysed by EffectorP 2.0 for effector prediction. The results showed that ChtBD1 was the potential effector (Table [Link], [Link]). Accordingly, we generated N‐ and C‐terminal truncated mutants to examine their ability for cell death induction by agroinfiltration in *N. tabacum* leaves (Figure 5a). The N‐terminal truncated mutant RsEG146^N70^ still maintained the cell death‐inducing activity, while the ChtBD1‐deleted mutant RsEG146^N22^ did not induce cell death. Likewise, the C‐terminal truncated mutants without ChtBD1 (RsEG146^C71^, RsEG146^C92^, and RsEG146^C306^) also lost necrosis‐inducing activity, except for RsEG146^C23^, which contains the ChtBD1 domain (Figure S7).

A small peptide or effector motif is often sufficient for necrosis induction (Yang et al. 2018a; Wei et al. 2020). To clarify the cell death‐inducing active peptide in ChtBD1 of RsEG146, we divided ChtBD1 into one β‐strand (S: Cys37‐Ser46), one α‐helix (H: Gly47‐Gly53) and two loops (L1: Gln23‐Pro36; L2: Gly54‐Pro70) based on its secondary structure (Figure S8). N‐ and C‐terminal truncated mutations were also used to find the functional peptide by agroinfiltration in *N. tabacum* leaves. The truncated mutants without the H part (RsEG146^C23‐46^, RsEG146^C47‐70^, RsEG146^C54‐70^) did not lead to cell death, which was all detected in *N. tabacum* leaves (Figure 5b), indicating that the helix structure in ChtBD1 of RsEG146 is vital for cell death induction.

The amino acid sequence of RsEG146^C47‐53^ is Gly‐Ser‐Gln‐Phe‐Cys‐Leu‐Gly. To further define the cell death‐inducing active amino acid residue in ChtBD1 of RsEG146, we preferentially mutated each amino acid residue to alanine except for two glycine residues, Gly47 and Gly53. The mutants were transiently expressed in *N. tabacum* leaves, but none of these mutants lost the cell death‐inducing activity (Figure S9). The truncated mutation was then used to find critical amino acid residues (Figure 5c). We found that mutant RsEG146^C23‐47^ (RsEG146^C23‐46^ followed by Gly47 only) and RsEG146^C47‐70^ (start with Gly47 and followed by RsEG146^C48‐70^) still maintained the necrosis‐inducing activity, while both RsEG146^C23‐46^ and RsEG146^C48‐70^ lost the function (Figure 5c). This result indicates that Gly47 is the critical residue in the chitin‐binding domain of RsEG146 that is sufficient for cell death elicitation.

### 2.6 Necrosis Function of Gly47 Can Be Incapacitated by Acidic Amino Acid and Proline Substitution

**FIGURE 6:**
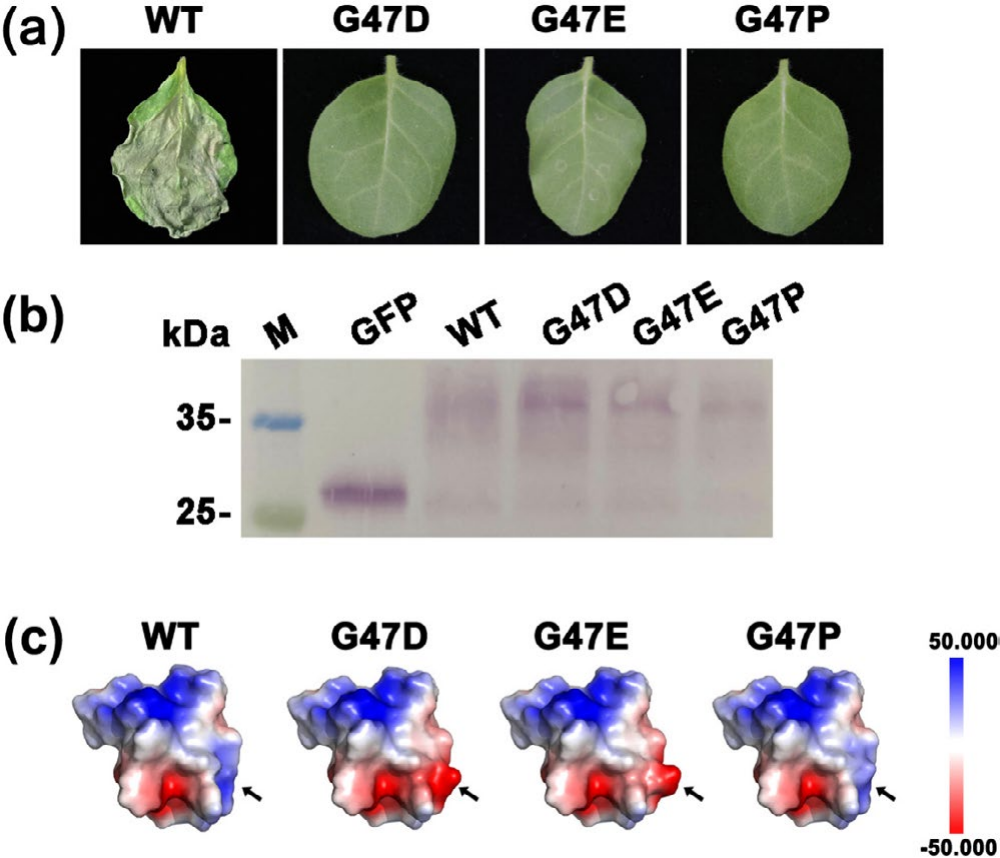
Necrosis function of Gly47 in RsEG146 can be incapacitated by acidic amino acid and proline substitution. (a) G47D, G47E and G47P did not induce cell death in *Nicotiana tabacum* . (b) Transient expression of GFP, wild type (WT), G47D, G47E and G47P in *N. tabacum* . (c) Protein electrostatic potential analysis of WT, G47D, G47E and G47P. The arrows indicates mutated sites. The mutations of G47D and G47E caused a change to negative charge (red), while G47P remained positively charge (blue).

To investigate the necrosis‐inducing mechanism of Gly47 in RsEG146, site‐saturation mutagenesis of Gly47 was performed, and the mutants were transiently expressed in *N. tabacum* leaves. Most of the mutants still induced cell death except for mutants G47D, G47E and G47P (Gly47 mutated to aspartic acid, glutamic acid, and proline, respectively) (Figure 6a,b; Figure S10). Surface charge analysis of wild‐type and mutant proteins showed that G47D and G47E changed the electric charge of the site from positive to negative. Although G47P remained positive in charge, the charge was lower than the wild type, and the structure of the substitutive residue was flatter than that of other mutants (Figure 6c; Figure S11).

### 2.7 RsEG146 Plays a Crucial Role to Pathogenicity of *R. solani*

**FIGURE 7:**
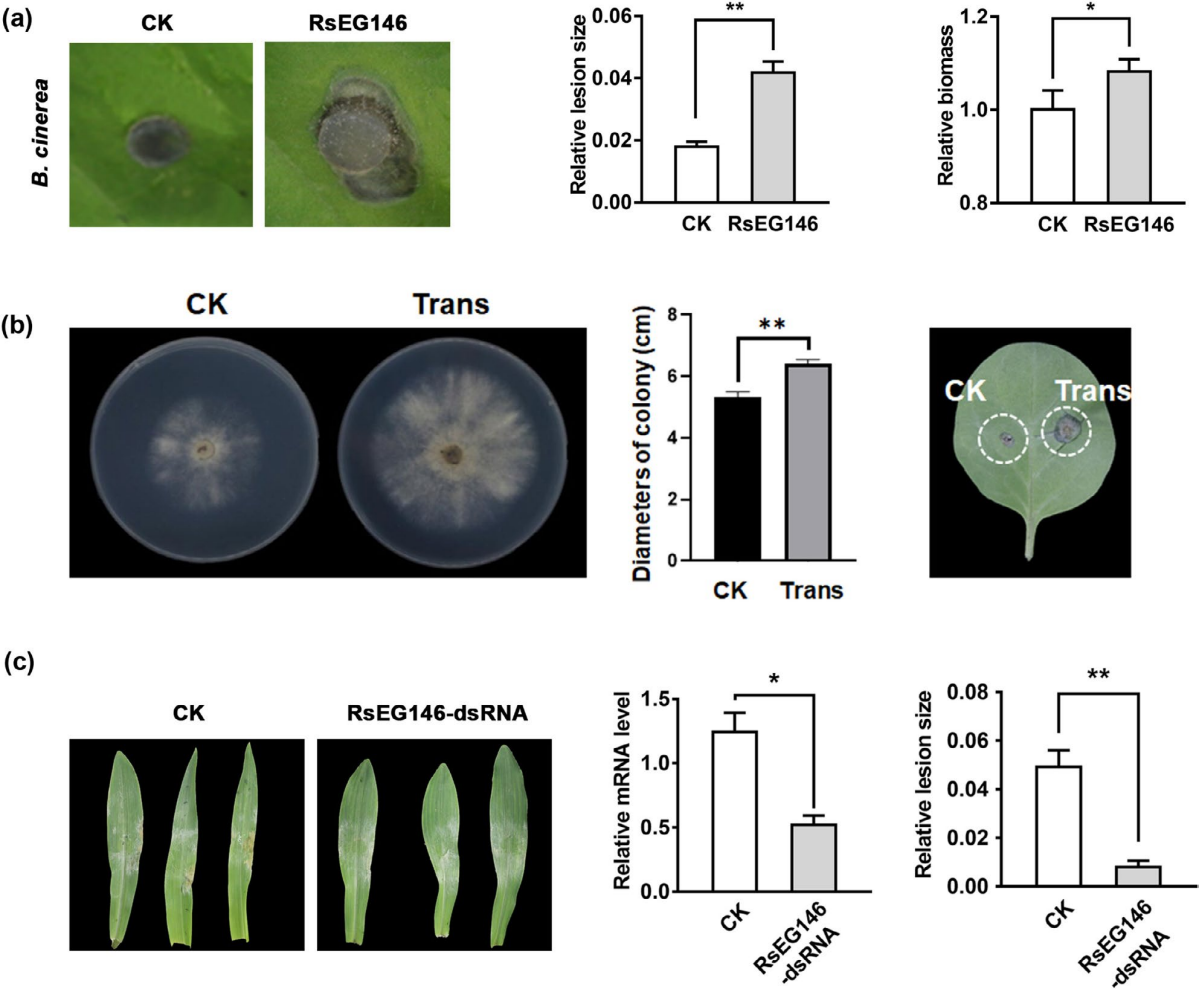
RsEG146 played crucial roles to pathogenicity of *Botrytis cinerea* and *Rhizoctonia solani*. (a) Transiently expressed *RsEG146* enhances the pathogenicity of *B. cinerea* on *Nicotiana tabacum* . *RsEG146* was transiently expressed on lower leaves, followed by inoculating a mycelial block of *B. cinerea* on upper leaves after 24 h. (b) Heterogeneous expressed *RsEG146* enhanced growth rate and pathogenicity of *B. cinerea* . (c) *RsEG146* silenced by spray‐induced gene silencing does not affect mycelial growth but reduces pathogenicity of *R. solani* . Symptom, lesion size and *RsEG146* expression level on *R. solani* ‐invaded *Zea mays* leaves. Column = mean ± SE (*p < 0.05, **p < 0.01).

To investigate the roles of RsEG146 during the pathogenic process, we transiently expressed RsEG146 in the lower leaves of *N. tabacum* , followed by inoculating *Botrytis cinerea* on the upper leaves. RsEG146‐treated tobacco plants exhibited larger lesion sizes and more biomass of *B. cinerea* (Figure 7a), indicating RsEG146 enhanced the pathogenicity of *B. cinerea* on tobacco. To further confirm this result, we performed heterogeneous expression of *RsEG146* in *B. cinerea* . Compared with the wild type of *B. cinerea* , the positive transformant grew faster and caused larger lesions than the wild type (WT) (Figure 7b).

We also silenced *RsEG146* expression by spray‐induced gene silencing (SIGS) (Chen et al. 2022). RsEG146‐dsRNA was first sprayed on potato dextrose agar (PDA) for 48 h, and gene expression, colony diameter, mycelial fresh weight, and dry weight of *R. solani* were determined. The results showed that *RsEG146* expression in *R. solani* was downregulated by RsEG146‐dsRNA but did not affect mycelial growth and biomass (Figure S12). Then RsEG146‐dsRNA was sprayed on 2‐week‐old maize leaves, followed by inoculating with *R. solani* . RsEG146‐dsRNA treatment prevented the pathogen from forming lesions, and the *RsEG146* expression level of *R. solani* in RsEG146‐dsRNA treated leaves was also downregulated (Figure 7c). The pathogen biomass in both control and treatment had no significant difference (Figure S12). These results showed that RsEG146 is crucial in *R. solani* AG1 IA pathogenicity.

### 2.8 *RsEG146* Induced Plant Defence Response

**FIGURE 8:**
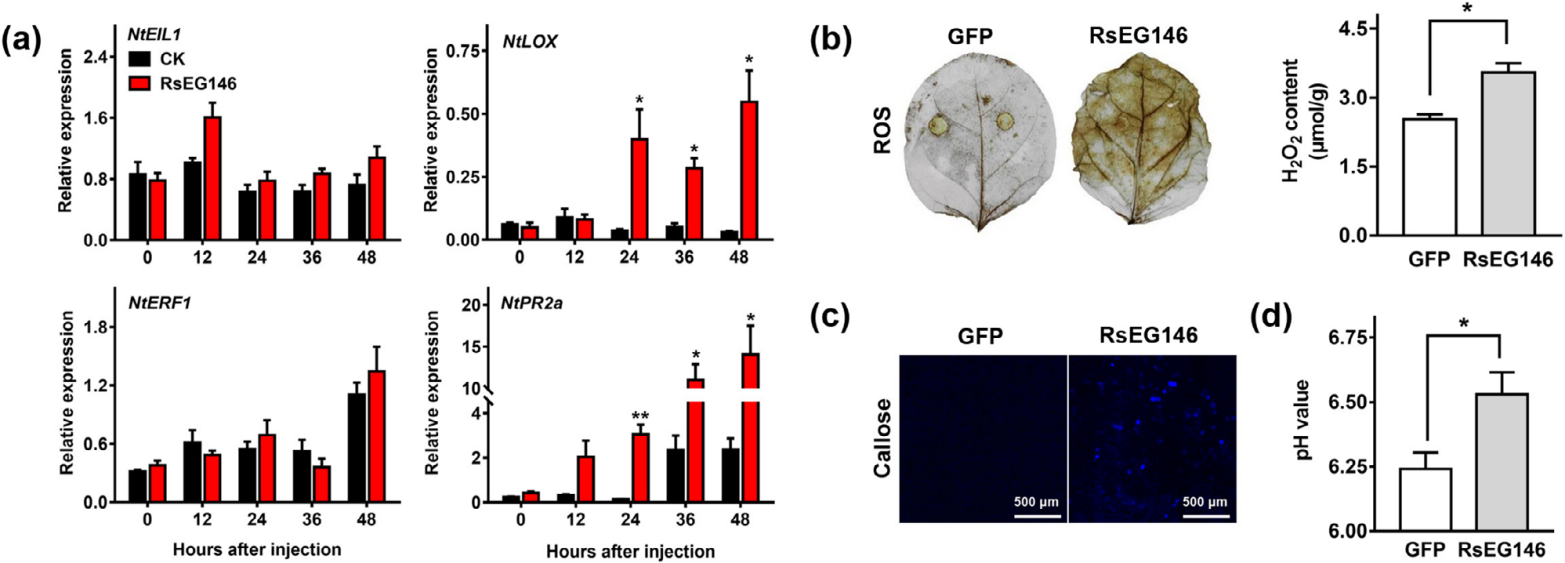
RsEG146 triggers plant immunity response in *Nicotiana tabacum* . (a) Expression of four plant immunity‐related genes in *N. tabacum* leaves transiently expressing *RsEG146*. Column = mean ± SE (*p < 0.05, **p < 0.01). (b) Accumulation of reactive oxygen species (ROS) and H_2_O_2_ content in *N. tabacum* leaves transiently expressing *RsEG146*. These experiments were replicated three times with three leaves per biological replicate. (c) Deposition of callose in *N. tabacum* leaves transiently expressing *RsEG146*. (d) Variation of pH in *N. tabacum* leaves transiently expressing *RsEG146*. Column = mean ± SE (*p < 0.05).

PTI mainly involves defence‐related gene expression, bursts of reactive oxygen species (ROS), callose deposition and alkalinisation (Ma et al. 2015a; Saijo et al. 2017). To identify whether RsEG146 elicits the defence response in *N. tabacum* , we used reverse transcription‐quantitative PCR (RT‐qPCR) to investigate the expression patterns of defence response‐related genes in *N. tabacum* , including pathogenesis‐related (PR) protein gene *NtPR2a*, transcription factor gene *NtERF1* (ethylene response factor 1), *NtEIL1* (ethylene‐insensitive like 1) and lipoxygenase gene *NtLOX*. Transient overexpression of *RsEG146* significantly induced the expression of *NtPR2a* and *NtLOX* genes compared with the control (Figure 8a). We also examined the ability of RsEG146 to activate ROS and hydrogen peroxide (H_2_O_2_) content in *N. tabacum* leaves transiently expressing *RsEG146*. The results showed that ROS, as well as the content of H_2_O_2_, were activated by RsEG146 at 2 dpi (Figure 8b). Furthermore, callose deposition was observed by blue fluorescence and higher pH values were observed in *N. tabacum* leaves transiently expressing *RsEG146* (Figure 8c,d). These results indicated that RsEG146 activated the *N. tabacum* defence response.

## 3 Discussion

*Rhizoctonia solani* is a necrotrophic pathogen that kills host plant cells and takes nutrients from dead tissue for colonisation and growth. The necrosis mechanism of *R. solani* is through the production of several virulence factors, mainly toxins and CWDEs (Oliver and Solomon 2010; Chen et al. 2017; Ren et al. 2017). Toxins produced by *R. solani* lead to the destruction of host cell structures, including membrane disintegration, mitochondria and chloroplast deformation and endoplasmic reticulum swelling (Vidhyasekaran et al. 1997; Chen et al. 2019; Frederick et al. 2016). CWDEs such as PGs, pectin methylgalacturonases and cellulases destroy the cell wall and promote the expansion of necrotic lesions (Chen et al. 2006; Chen et al. 2017). Necrotrophic pathogens also secrete effector proteins as host‐specific or host‐selective toxins to promote tissue or cell necrosis, playing important roles in host–pathogen interactions (Anderson et al. 2017). Some toxins and CWDEs from *R. solani* can be effectors that activate the host defence response (Zuo et al. 2014; Ma et al. 2015b). A GH45 member enzyme EG1 of *R. solani* as a PAMP combines the functions of cell wall degradation and induction of defence responses, which are independent of each other (Ma et al. 2015b). In our study, a GH16 member effector RsEG146 from *R. solani* has a little cellulose degrading activity (Figures S5 and S6) but induces strong host cell death and defence response, suggesting that more than one kind of CWDEs are effectors in *R. solani* and they participate in the necrotic process, independent of their cell wall‐degrading activity.

GH16 family members play crucial roles in plant and fungal cell wall degrading or remodelling, fungicide development and biomass degradation (Meng et al. 2016; McGregor et al. 2017; Fang et al. 2019; Viborg et al. 2019), even acting as effectors and elicitors of plant defence. *B. cinerea* , a necrotrophic plant pathogen, produces a GH16 family member BcCrh1 with a single conserved domain that is not only important for fungal development but also induces plant cell death and defence responses. The cell death‐inducing activity of BcCrh1 has no relationship with its plant cell wall‐degrading activity. The site is a residue region and is located in the catalytic domain (Bi et al. 2021). In our study, the necrotrophic fungal pathogen *R. solani* possesses a GH16 enzyme RsEG146 with two main conserved domains that have a similar function to BcCrh1. Compared with BcCrh1, the key site of cell death induction in RsEG146 is a single amino acid, Gly47, located in a noncatalytic domain, ChtBD1, far from the enzymatic activity domain, LamG. Furthermore, *RsEG146* silenced by SIGS did not affect fungal development. Our study enriched knowledge of the class of necrosis‐inducing proteins in GH16 family, and further demonstrated that members of this family are important participants in host–pathogen interactions. We also found the active site of cell death induction of RsEG146. Whether the cell death‐inducing active sites of the GH16 family have certain rules on sequence or structure needs further study.

The chitin‐binding domain (ChtBD) is a main structure of chitinase that plays important roles in helping the enzyme to increase substrate affinity and enhance catalytic efficiency (Suginta et al. 2016; Oyeleye and Normi 2018). It exists in the N‐terminal of class I and IV chitinases that are members of the glycoside hydrolase family 19 (GH19) (Kezuka et al. 2010), and this domain is involved in improving the antifungal activity of chitinases from plants and animals (Yokoyama et al. 2009; Chen et al. 2018). ChtBD is also referred to as hevein or hevein‐like protein and is grouped into carbohydrate‐binding module family 18 (CBM18), a family including several plant lectins composed of one or more hevein domain(s) (Oguri et al. 2008). Hevein and hevein‐like proteins inhibit the growth of chitin‐containing fungi and protect plants from infection by a wide range of fungal pathogens (Van Parijs et al. 1991; Koo et al. 2002). The antifungal activity of these antimicrobial peptides (AMPs) can be affected by amino acid residues, net charge, hydrophobicity, amphipathicity and other factors such as pH, temperature and metal ions (Li et al. 2021). In our study, though the similar structure domain ChtBD1 was located at the N‐terminal of RsEG146, interestingly, it has the completely opposite function. Differing from hevein‐like AMPs of plants that inhibit fungi growth and protect plants from pathogen attack, ChtBD1 of RsEG146 is a fungal protein domain that elicits the plant defence response. It also destroys the plant cell transmembrane potential and pH gradient, as plant hevein‐like AMPs cause on fungi. The function of ChtBD1 is affected by amino acid residues and net charge. We suggest that ChtBD1 of RsEG146 is a fungal hevein‐like ‘antiplant’ domain, leading to plant cell death and further benefitting the colonisation and growth of necrotrophic pathogens.

Proteins, as a type of polyelectrolyte, exhibit structural diversity due to different compositions of amino acids. The biological functions of proteins are largely dependent on their unique structures, which are determined by complex interactions between residues such as dispersion forces, hydrophobic and hydrophilic interactions and van der Waals forces (Ainis et al. 2019; Zhou and Pang 2018). As demonstrated by Anfinsen's denaturation experiment, the three‐dimensional structure of a protein is directly determined by its primary structure, and the stability of the protein is crucial for its activity. Many studies have shown that the presence of individual amino acids determines whether a protein has enzymatic activity; for example, the two cysteine residues at positions 25 and 126 in RsIA_CtaG/Cox11 are essential for its immunosuppressive activity (Zhang et al. 2024); the conserved serine residue at position 120 in the DPBB folding domain of RsRlpA is crucial for its inhibition (Charova et al. 2020); the N‐terminal P62, W63, and D67 and the C‐terminal C204 and R206 in the active site of EG1 play a critical role (Guo et al. 2021). In this study, we found that the active key site of RsEG146 that triggers host cell death is G47. When the site was mutated, only substitutions with acidic amino acids and proline lost the ability to induce cell death. The results of analysing the surface charge of RsEG146 after mutation show that the surface charge distribution of RsEG146 with G47 mutated to an acidic amino acid is obviously different from that of other mutants. Whether the changes in RsEG146‐induced cell death activity are influenced by its surface charge distribution remains to be further studied.

In this study, we identified a GH16 family secreted protein RsEG146 in the necrotrophic fungus *R. solani* AG1 IA as an effector. It induced cell death and defence responses in *N. tabacum* leaves. We also confirmed the key necrosis‐inducing site Gly47 in the chitin‐binding domain of RsEG146. Nevertheless, the precise molecular mechanisms of RsEG146 in host– *R. solani* interactions, including which proteins RsEG146 interacts with and how RsEG146 coordinates the pathogenesis and induction of resistance, still remain to be studied.

## 4 Experimental Procedures

### 4.1 Strains, Plant Materials and Growth Conditions

*Rhizoctonia solani* AG1 IA strain YN‐7 was used in this study. *R. solani* was cultured on PDA (20% potato, 2% glucose, 1.5% agar) at 28°C. *Agrobacterium tumefaciens* GV3101 was grown on Luria Bertani (LB) medium (0.5% yeast extract, 1% tryptone, 1% NaCl) at 28°C. The yeast strain YTK12 was cultivated on YPDA medium (1% yeast extract, 2% tryptone, 2% glucose, 2% agar, 0.003% adenine sulphate) at 28°C. The concentrations of antibiotics used in this study were as follows: kanamycin 100 μg/mL, ampicillin 100 μg/mL, and rifampin 25 μg/mL. *B. cinerea* was cultured on PDA for 5 days at 25°C; mycelial plugs with a diameter of 6 mm were punched at the edge of the colony used for inoculating tobacco leaves. *N. tabacum* ‘Samsung’ and *Zea mays* were grown in a greenhouse under a 12/12 h photoperiod at daytime and nighttime, respectively, and the temperature was kept at 26°C. All data are based on at least five repeats.

### 4.2 Gene Cloning and Sequence Analysis

gDNA and total RNA of *R. solani* AG1 IA were extracted by following the manufacturer's instructions of the DNA extraction kit (Solarbio) and the RNA extraction kit (Vazyme), respectively. cDNA based on the total RNA of *R. solani* was obtained by using the TransScript One‐Step gDNA Removal and cDNA Synthesis SuperMix kit (TransGen). Full‐length and ORF of the *RsEG146* gene were amplified based on gDNA and cDNA, respectively, with Phanta Max Super‐Fidelity DNA Polymerase (Vazyme).

The amino acid sequence of RsEG146 was used as a BLAST query in the National Center for Biotechnology Information (NCBI) database (https://www.ncbi.nlm.nih.gov/). The top 100 sequences in BLAST searches were downloaded from NCBI, and the cladograms were built with MEGA X using the neighbour‐joining method and were modified by using iTOL (https://itol.embl.de/). The signal peptide of RsEG146 was predicted by SignalP (http://www.cbs.dtu.dk/services/SignalP/). The transmembrane domain was predicted by the TMHMM Server (http://www.cbs.dtu.dk/services/TMHMM/). Conserved Domains‐search in NCBI was used to predict the domains of RsEG146 (https://www.ncbi.nlm.nih.gov/Structure/cdd/wrpsb.cgi). Three‐dimensional structures of RsEG146 domains were built by using SWISS MODEL (https://swissmodel.expasy.org/).

For electrostatic potential analysis, three‐dimensional structures of RsEG146 ChtBD1 domains were built by using SWISS MODEL. The structures of the mutants were generated by using the Mutagenesis function in PyMOL software. Surface charge was calculated for each structure by using protein contact potential in the Vacuum Electrostatics function, followed by adjusting the scale range from −50 to 50 in PyMOL.

### 4.3 Plasmid Construction

Restriction enzymes (Thermo Scientific) were used according to the manufacturer's instructions. Full‐length and ORF of *RsEG146* were cloned in a Blunt Simple cloning vector based on the manufacturer's instructions (TransGen). PCR products of transiently expressed genes were digested with BamHI and XhoI, purified with a gel purification kit (Omega) and inserted into the pMD1‐35S‐GFP expression vector. The SP sequence was amplified by digesting with EcoRI and XhoI and inserted into the pSUC2 vector (Jacobs et al. 1997). All constructs were sequenced for confirmation. Primers used in this study are listed in Table S2.

### 4.4 Truncated Mutant Construction and Site‐Directed Mutagenesis

Truncated mutant genes of *RsEG146* for transient expression in *N. tabacum* leaves were constructed using different strategies. For N‐terminal truncated mutants, genes were directly amplified by using different primer pairs. For C‐terminal truncated and enzymatic active site deletion mutants, mutant genes were obtained by reverse amplification occurring in the *RsEG146*‐linked cloning vector. Site‐directed mutagenesis was performed using the Fast Mutagenesis System kit. Primer pairs are listed in Table S2.

### 4.5 Transient Expression of *RsEG146* in *N. tabacum* and Subcellular Localisation

pMD1‐35S‐GFP vectors were transformed into *A. tumefaciens* GV3101. The strains were cultured in LB medium with kanamycin and rifampicin at 28°C, 200 rpm overnight. The bacterial suspensions were collected and resuspended in MES buffer (10 mM MES [pH 5.6], 10 mM MgCl_2_, 150 μM acetosyringone) to the optical density (OD) at 600 nm of 0.6. Infiltration into *N. tabacum* leaves was performed with needleless injectors after 1 h incubation at 28°C. Empty pMD1‐35S‐GFP vector was used as a control. Cell death in leaves was observed 2 days after injection. For subcellular localisation, *A. tumefaciens* containing pCAMBIA1300‐mCherry‐HDEL was used as an ER marker and was co‐infiltrated into tobacco leaves. GFP and mCherry fluorescence were observed using confocal laser microscopy (FV1000; Olympus) with 488 and 561 nm excitation lasers, respectively.

### 4.6 Function Validation of the SP

We performed a yeast secretion assay to identify the function of the predicted SP of RsEG146 according to Jacobs et al. (1997) and Gui et al. (2017). pSUC2 vectors were transformed into the yeast strain YTK12 with the Yeast Transformation Kit (Coolaber). The transformed yeast was cultured on YPDA, CWD−W medium (0.075% tryptophan dropout supplement, 0.67% yeast nitrogen base without amino acid, 2% sucrose, 0.1% glucose and 2% agar [pH 5.8]) and YPRAA medium (1% yeast extract, 2% peptone, 2% raffinose, 2 μg/mL antimycin A, 2% agar) plates at 28°C. The invertase enzymatic activity was verified by the reduction of TTC to insoluble red‐coloured triphenylformazan as described in Liu et al. (2013). The secretion signal of α‐factor from the eukaryotic expression vector pPIC9K and the non‐functional N‐terminus of Mg87 from *M. grisea* were set as a positive control and a negative control, respectively.

### 4.7 Protein Extraction and Immunoblotting

Tobacco leaves infiltrated by *Agrobacterium* were harvested at 48 h, then ground in liquid nitrogen. The powders were mixed with prechilled protein extraction buffer (NP‐40; Beyotime) containing 1 mM phenylmethanesulfonyl fluoride (PMSF). The mixture was incubated overnight at 4°C and centrifuged at 10,625*g* at 4°C for 10 min. The supernatant was mixed with 5 × SDS‐PAGE loading buffer, then boiled in water for 10 min. The extracted proteins were separated by SDS‐PAGE at 140 V, 110 mA, and electroblotted onto PVDF membranes (0.22 μm) at 15 V, 20 mA for 60 min. The transferred membranes were blocked with 5% defatted milk in TBST buffer (20 mM Tris–HCl, pH 7.5; 150 mM NaCl; 0.05% Tween 20) for 1 h at room temperature. The membranes were washed three times followed by adding primary antibodies, then incubated at 4°C overnight. The membranes were washed with TBST buffer every 10 min for three times. Then the membranes were incubated in secondary antibody (alkaline phosphatase‐conjugated goat anti‐rabbit IgG; BBI Life Science) for 1 h at room temperature. After that, the membranes were washed three times with TBST buffer, then the proteins were detected by using BCIP/NBT in darkness.

### 4.8 Heterogeneous Expression of RsEG146 in *B. cinerea*

The myceliual pellets of *B. cinerea* were inoculated in 50 mL potato dextrose broth (PDB) and shaken for 48 h. The mycelia were washed with water three times, then cut with sterile scissors and placed in the enzymolysis solution at 28°C and 100 rpm for 4–6 h. The enzymolysis solution was filtered with sterile filter paper, and the filtrate was collected. This was centrifuged at 1845*g* at 4°C for 10 min, the supernatant was discarded, and STC buffer was added to resuspend the protoplasts. Full‐length *RsEG146* (0.2 μg/μL) was added to 125 μL of protoplasts and incubated on ice for 30 min. One millilitre of PTC buffer was gradually added and mixed, then centrifuged at 4°C and 4722 *g* for 5 min. The supernatant was discarded, and the precipitate was promptly placed on ice and resuspended with 500 μL of STC buffer. After shaking at 28°C for 12–16 h, it was mixed with TB3 medium containing hygromycin B. After incubation at 28°C for 16–18 h, another layer of TB3 medium containing 300 μg/mL hygromycin B was overlaid, followed by incubating at 28°C. The grown colonies were transferred to new TB3 medium three to five times. The transformants were verified by sequencing.

### 4.9 SIGS in *R. solani*

Synthesis of dsRNAs in vitro was performed as in the previous study (Chen et al. 2022). Primers used for synthesising dsRNAs were listed in Table [Link], [Link]. The PDA in Petri dishes was covered with sterile cellophane, and 100 μL dsRNA at 50 ng/μL, with sterile water as control. Mycelial plugs 6 mm in diameter from the colony edge of *R. solani* were cultured in the centre of PDA for 36 h. After being cultured at 28°C for 2 days, colony diameters, fresh weight and dry weight of mycelia were determined. Then mycelia were collected from the cellophane for RNA extraction, which was then used to detect the expression levels of genes in *R. solani* , using *RsGAPDH* as the reference gene. Relative mRNA levels were counted as the target gene expression level divided by the reference gene expression level. Synthetic dsRNAs with a final concentration of 50 ng/μL were sprayed on the surface of 2‐week‐old *Z. mays* leaves, followed by inoculating mycelial plugs. After 36 h, leaves were collected for relative lesion size measurement, gene expression level detection and mycelial biomass determination. Relative lesion sizes were counted as the ratio of lesion area to whole leaf area measured by using Photoshop software. Relative biomass was counted as *RsGAPDH* expression level divided by host reference gene expression level (*NtGAPDH* for *N. tabacum* and *ZmUBQ9* for maize).

### 4.10 Conductivity Measurement, pH Variation, Trypan Blue Staining, ROS Burst, H_2_O_2_ Concentration and Callose Deposition

We measured the conductivity of leaves as a marker of cell death. *N. tabacum* leaves were collected 48 h after infiltration and were punched into small pieces with a 6 mm hole puncher. Small pieces were immersed in sterile water at room temperature for 1 h, then the conductivity of the solution was measured and the value noted as R_i_. Each solution was boiled for 5 min, then cooled to room temperature. We measured the conductivity of the solution again and noted the value as *R*_t_. Relative conductivity *R*_0_ (%) was calculated according to the formula *R*_0_ = *R*_i_/*R*_t_ × 100. pH variation was prepared by grinding *N. tabacum* leaves in liquid nitrogen and soaking the powders in sterile water. The mixture was mixed by a vortex oscillator and centrifuged at 10,625*g* at room temperature for 10 min. The pH of the liquid supernatant was measured by a pH meter. Trypan blue staining, ROS burst, H_2_O_2_ concentration, and callose deposition detection were performed as described previously (Wei et al. 2020). All experiments were repeated three times.

## Conflicts of Interest

The authors declare no conflicts of interest.

## Supporting information


Figure S1.

Figure S2.

Figure S3.

Figure S4.

Figure S5.

Figure S6.

Figure S7.

Figure S8.

Figure S9.

Figure S10.

Figure S11.

Figure S12.

Table S1.

Table S2.

## Data Availability

The data that support the findings of this study are available from the corresponding author upon reasonable request.
