# Supplementary material for: Glycoside Hydrolase Family 16 Enzyme RsEG146 From Rhizoctonia solani AG1 IA Induces Cell Death and Triggers Defence Response in Nicotiana tabacum
Source: Mol Plant Pathol. 2025 Mar 17;26(3):e70075. doi: 10.1111/mpp.70075 (PMC11911542; doi:10.1111/mpp.70075)
Supplement: Supplementary file 1 — Figure S1. [file MPP-26-e70075-s007.docx]

**
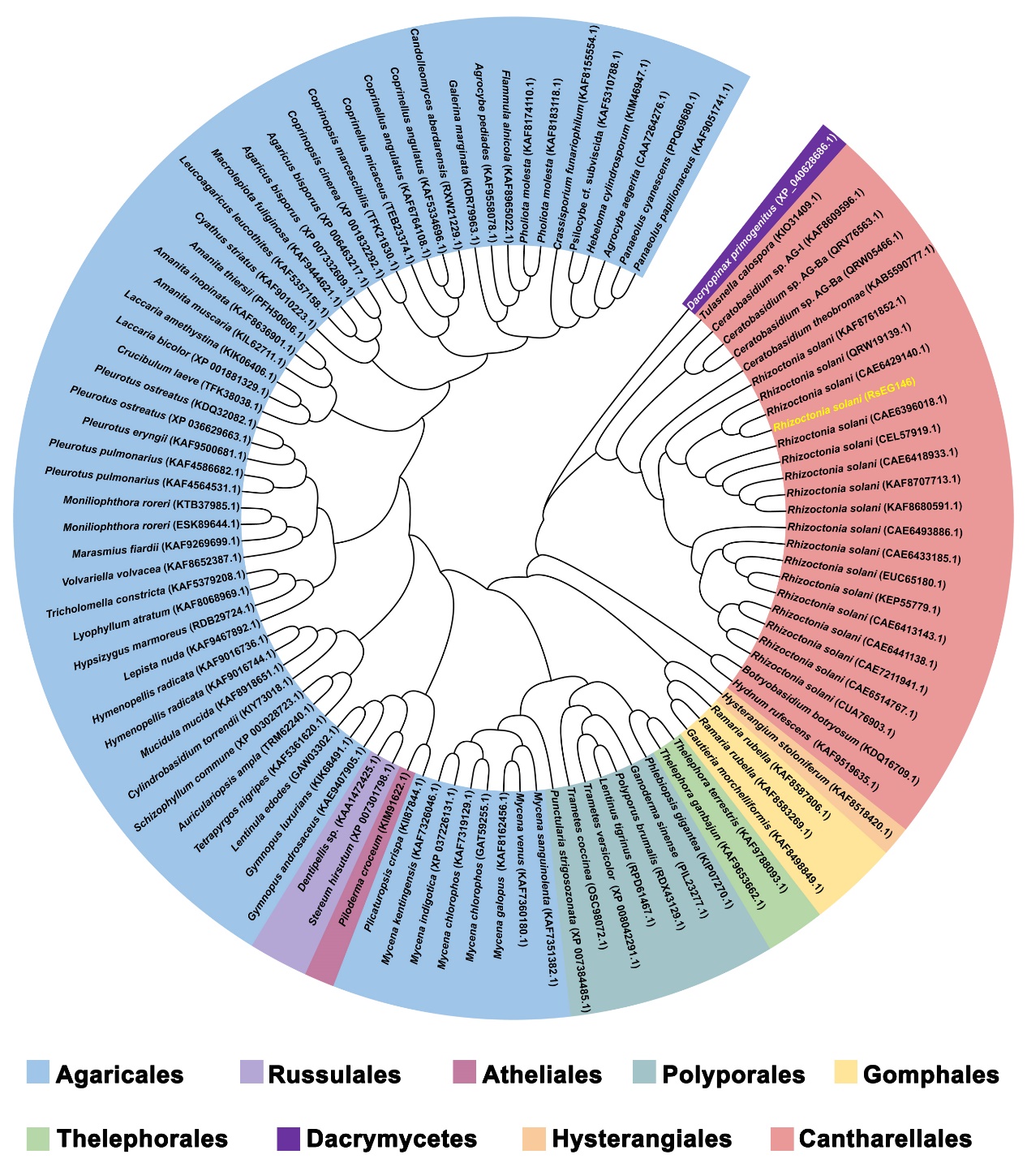
Figure Figure S1 Cladogram of RsEG146 and glycoside hydrolase family 16 members in other Basidiomycota fungi.**
