# Supplementary material for: Glycoside Hydrolase Family 16 Enzyme RsEG146 From Rhizoctonia solani AG1 IA Induces Cell Death and Triggers Defence Response in Nicotiana tabacum
Source: Mol Plant Pathol. 2025 Mar 17;26(3):e70075. doi: 10.1111/mpp.70075 (PMC11911542; doi:10.1111/mpp.70075)
Supplement: Supplementary file 2 — Figure S2. [file MPP-26-e70075-s006.docx]

**
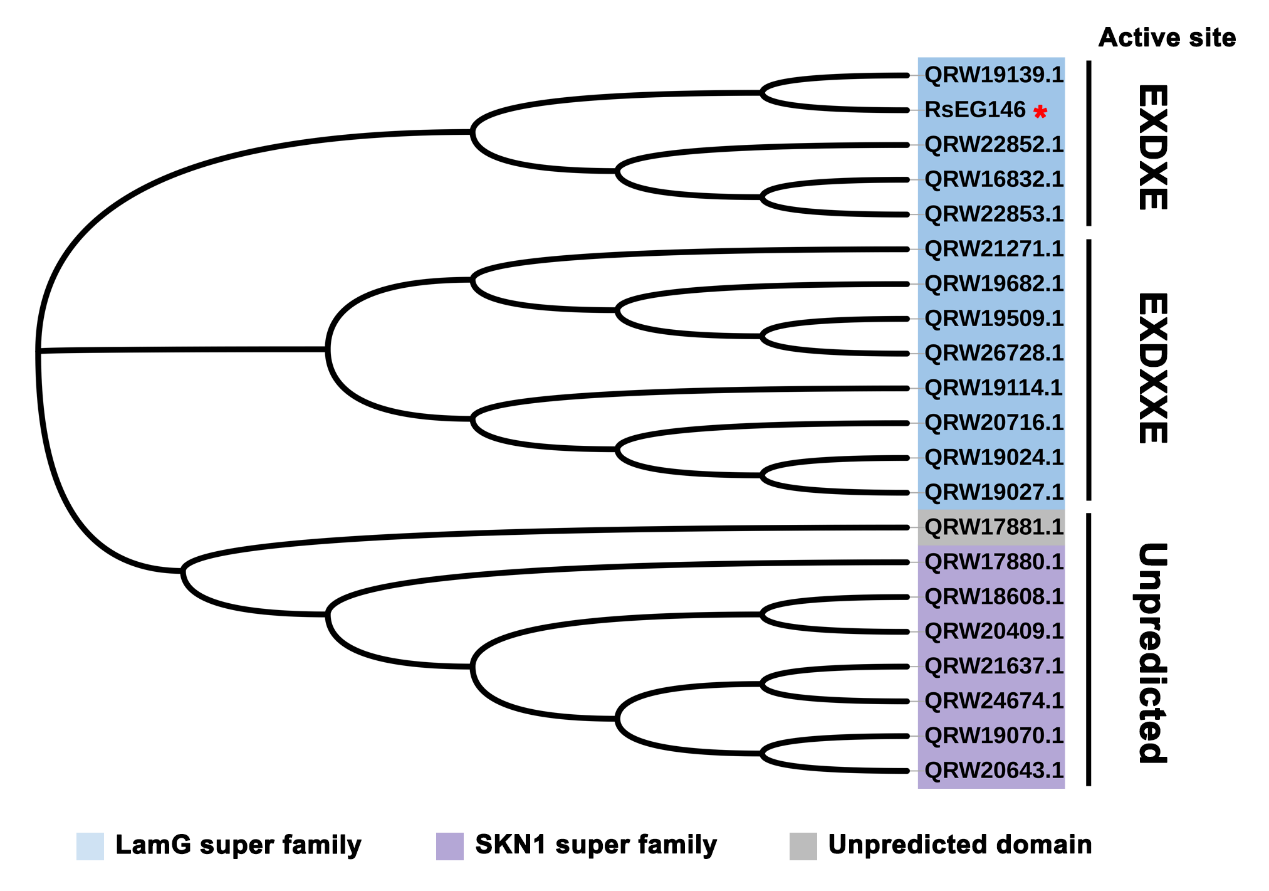
**

**Figure S2 Cladogram of RsEG146 and other glycoside hydrolase family 16 members in *Rhizoctonia solani*.**
