# Supplementary material for: Glycoside Hydrolase Family 16 Enzyme RsEG146 From Rhizoctonia solani AG1 IA Induces Cell Death and Triggers Defence Response in Nicotiana tabacum
Source: Mol Plant Pathol. 2025 Mar 17;26(3):e70075. doi: 10.1111/mpp.70075 (PMC11911542; doi:10.1111/mpp.70075)
Supplement: Supplementary file 3 — Figure S3. [file MPP-26-e70075-s005.docx]

**
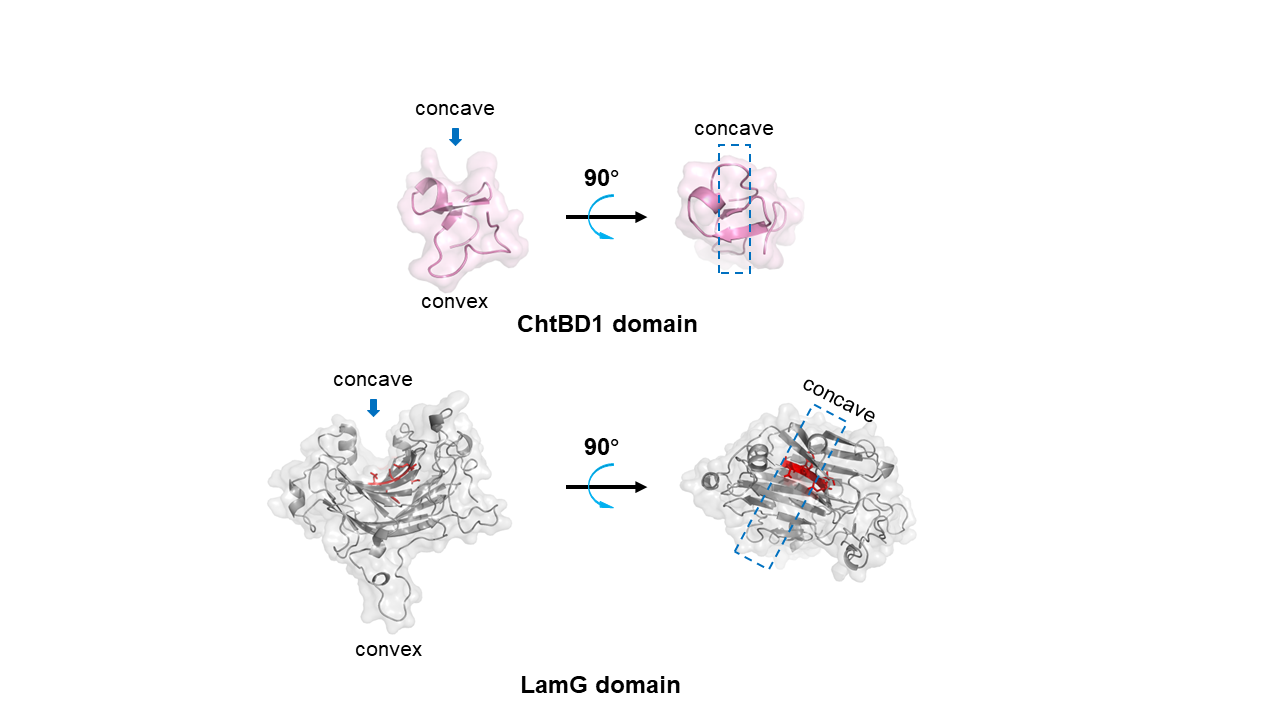
**

**Figure S3 Three-dimension structure of two main domains in RsEG146 constructed by using SWISS-MODEL.** The 3D structure models of ChtBD1 and LamG in RsEG146 were built by comparing with a plant source of chitinase (PDB ID: 6LNR) and a fungal transglycosylase (6IBU), respectively. Active side was colored as red in LamG domain.
