# Supplementary material for: Glycoside Hydrolase Family 16 Enzyme RsEG146 From Rhizoctonia solani AG1 IA Induces Cell Death and Triggers Defence Response in Nicotiana tabacum
Source: Mol Plant Pathol. 2025 Mar 17;26(3):e70075. doi: 10.1111/mpp.70075 (PMC11911542; doi:10.1111/mpp.70075)
Supplement: Supplementary file 4 — Figure S4. [file MPP-26-e70075-s010.docx]

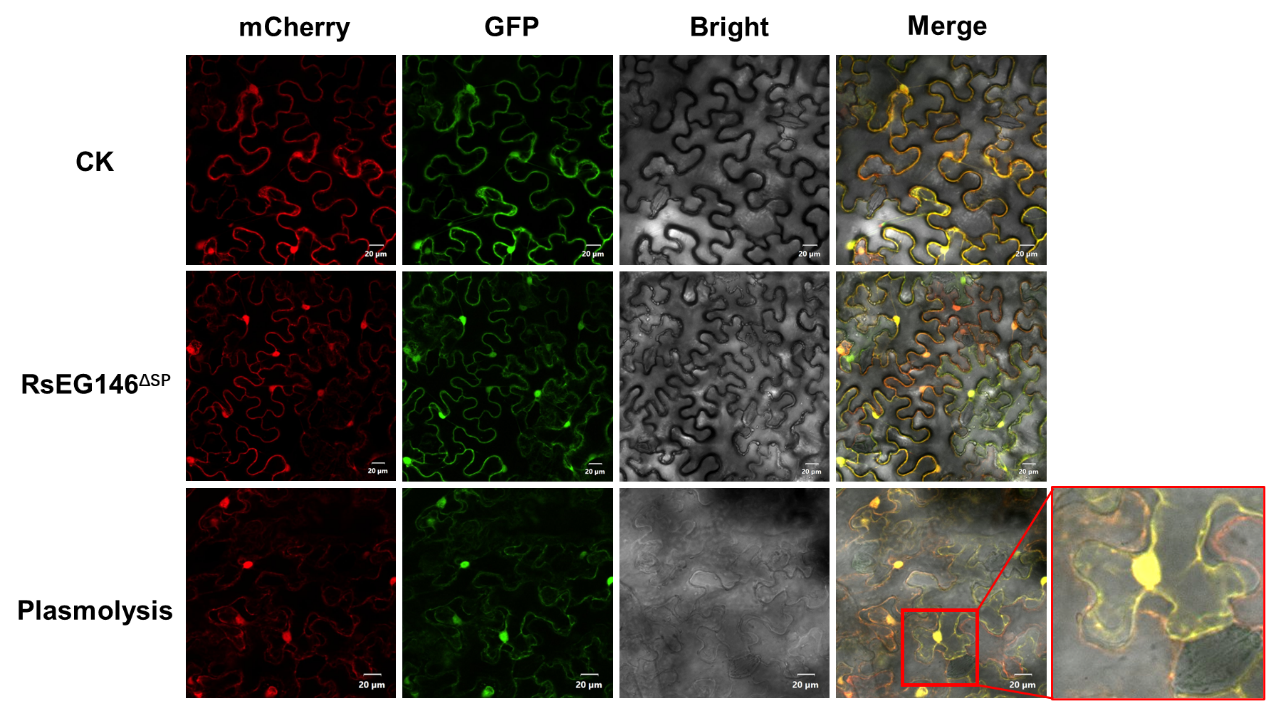


**Figure S4 The co-localization and plasmolysis assay of transient expressed RsEG146^ΔSP^ in tobacco leaves using nuclear marker (H2B-mCherry) and plasma membrane marker (PM-mCherry).** For subcellular localization, *A. tumefaciens* containing p2300-35S-H2B-mCherry was used as nuclear marker, pCAMBIA1300-35S-PM-mCherry was used as plasma membrane marker.
