# Supplementary material for: Glycoside Hydrolase Family 16 Enzyme RsEG146 From Rhizoctonia solani AG1 IA Induces Cell Death and Triggers Defence Response in Nicotiana tabacum
Source: Mol Plant Pathol. 2025 Mar 17;26(3):e70075. doi: 10.1111/mpp.70075 (PMC11911542; doi:10.1111/mpp.70075)
Supplement: Supplementary file 5 — Figure S5. [file MPP-26-e70075-s004.docx]

**
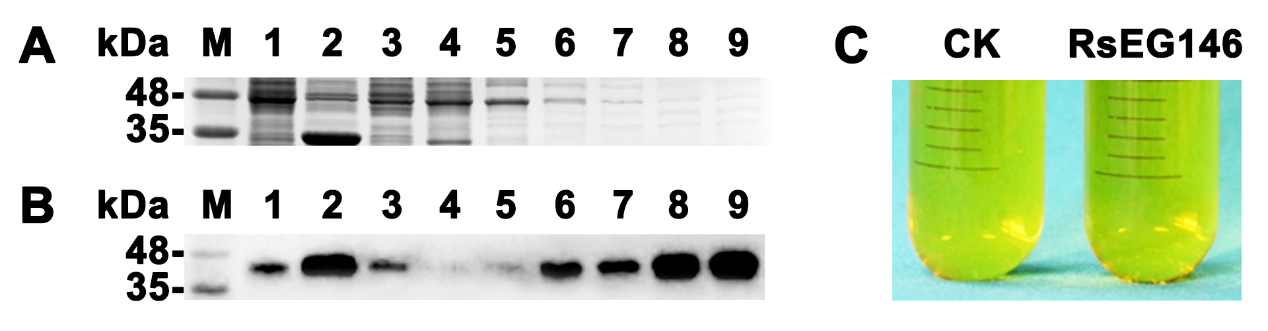
**

**Figure S5 Prokaryotic expression product of RsEG146 has no cell wall degrading enzyme activity.** RsEG146 gene was connected to prokaryotic expression plasmid pET-28a (digested with *EcoR*I and *Hind*III). The constructed plasmid was transformed into *Escherichia coli* Rosetta (DE3) strain. Screening was performed by using LB medium containing 0.1% kanamycin. 1000 mL of LB medium (supplemented with 0.1% kanamycin) was inoculated with 10 ml of pre-cultured *E. coli* Rosetta (DE3) strain containing the constructed plasmid. The cultures were incubated at 37 °C with 200 rpm shaking until they reached OD_600_ = 0.6. The culture was cooled to 28 °C, IPTG (isopropyl-β-D-thiogalactoside) was added to a final concentration of 1 mM, and the cultures were incubated with shaking for 17 h. *E. coli* cells were harvested by centrifugation at 12,000 rpm for 10 min at 4 °C. The pellet was resuspended in PBS (50 mM sodium phosphate, 300 mM sodium chloride with 10 mM imidazole; pH 7.4). The cells were busted by sonication, then centrifuged at 12,000 rpm for 10 min at 4 °C. The supernatant was subjected to a protein purification assay using BeaverBeads™ His-tag Protein Purification Kit (BeaverBeads, China) following the instruction. **A**, SDS-PAGE; **B**, Western blot. 1, Supernatant; 2, Precipitate; 3, Eluent; 4-9, 10mM, 50mM, 80mM, 100mM, 200mM and 300mM of imidazole. The primary antibody is Mouse His tag-mAb and the secondary antibody is Goat Anti-Mouse IgG-HRP (Abmart); **C**, RsEG146 enzymic activity detected by using DNS method (Miller, 1959).

Reference:

Miller, G. L. (1959) Use of dinitrosalicylic reagent for determination of reducing sugar. *Anal. Chem.*, 31, 426-428.
