# Supplementary material for: Glycoside Hydrolase Family 16 Enzyme RsEG146 From Rhizoctonia solani AG1 IA Induces Cell Death and Triggers Defence Response in Nicotiana tabacum
Source: Mol Plant Pathol. 2025 Mar 17;26(3):e70075. doi: 10.1111/mpp.70075 (PMC11911542; doi:10.1111/mpp.70075)
Supplement: Supplementary file 6 — Figure S6. [file MPP-26-e70075-s013.docx]

**
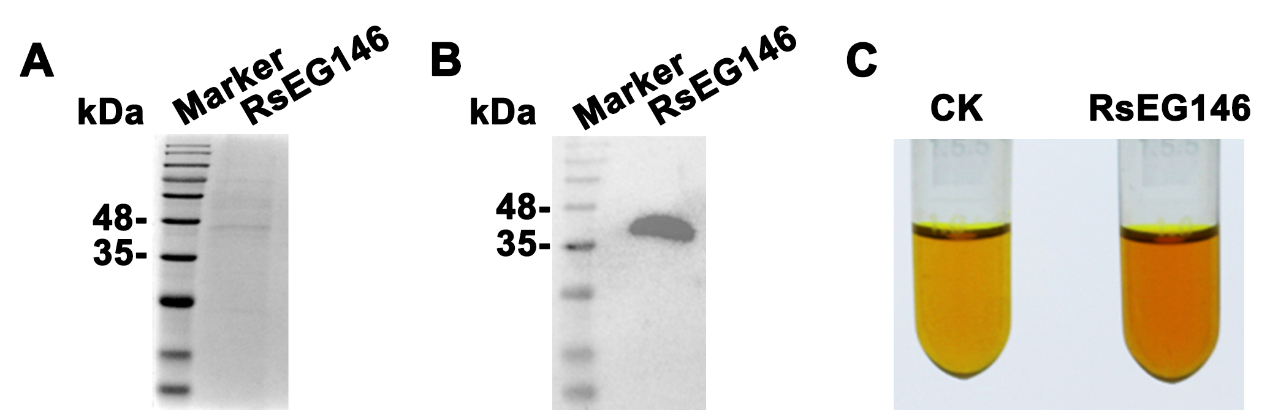
**

**Figure S6 Eukaryotic expression product of RsEG146 has a little cell wall degrading enzyme activity.** RsEG146 gene was connected to eukaryotic expression plasmid pPIC9K (digested with *EcoR*I and *Not*I). The constructed plasmid was transformed into *Pichia pastoris* GS115 strain. Screening was performed by using YPD medium containing 3 mg/mL and 4mg/mL geneticin (G418). Protein induction and purification were performed according to Ma et al. (2015). The rough extraction of RsEG146 was subjected to a protein purification assay using BeaverBeads™ His-tag Protein Purification Kit (BeaverBeads, China) following the instruction. **A**, SDS-PAGE; **B**, Western blot. The primary antibody is Mouse His tag-mAb, and the secondary antibody is Goat Anti-Mouse IgG-HRP (Abmart); **C**, RsEG146 enzymic activity detected by using DNS method (Miller, 1959).

Reference:

Miller, G. L. (1959) Use of dinitrosalicylic reagent for determination of reducing sugar. *Anal. Chem.*, 31, 426-428.
