# Supplementary material for: Glycoside Hydrolase Family 16 Enzyme RsEG146 From Rhizoctonia solani AG1 IA Induces Cell Death and Triggers Defence Response in Nicotiana tabacum
Source: Mol Plant Pathol. 2025 Mar 17;26(3):e70075. doi: 10.1111/mpp.70075 (PMC11911542; doi:10.1111/mpp.70075)
Supplement: Supplementary file 7 — Figure S7. [file MPP-26-e70075-s008.docx]

**
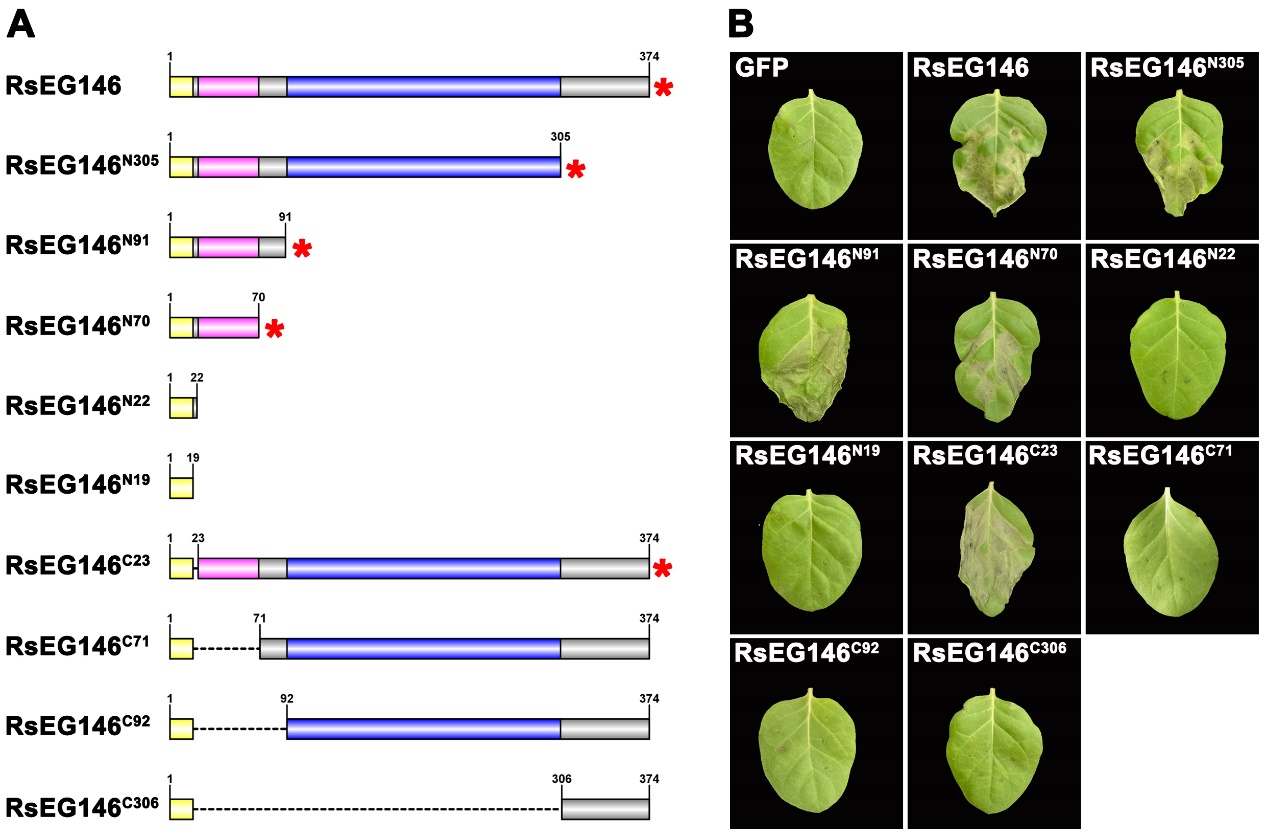
**

**Figure S7 Chitin binding domain ChtBD1 is sufficient for inducing cell death in *N. tabacum*. A,** Schematic presentation of total examined constructs. Mutants with necrotic activity were indicated by red stars. **B,** Analysis of protein domain inducing cell death by transiently expressing various truncated mutants in *N. tabacum* leaves.
