# Supplementary material for: Glycoside Hydrolase Family 16 Enzyme RsEG146 From Rhizoctonia solani AG1 IA Induces Cell Death and Triggers Defence Response in Nicotiana tabacum
Source: Mol Plant Pathol. 2025 Mar 17;26(3):e70075. doi: 10.1111/mpp.70075 (PMC11911542; doi:10.1111/mpp.70075)
Supplement: Supplementary file 8 — Figure S8. [file MPP-26-e70075-s002.docx]

**
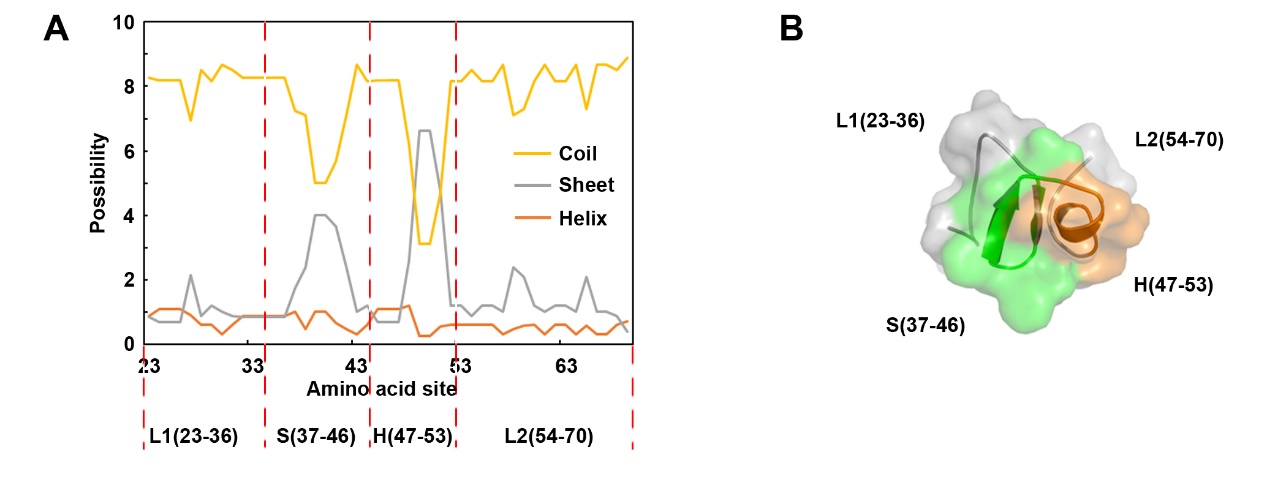
**

**Figure S8 Secondary structure analysis of RsEG146^C23-70^. A,** Secondary structure of RsEG146^C23-70^ analyzed by DNAMAN. **B**, Homologous model of RsEG146^C23-70^ constructed by SWISS-MODEL. L1, loop 1; S, sheet; H, helix; L2, loop 2.
