# Supplementary material for: Glycoside Hydrolase Family 16 Enzyme RsEG146 From Rhizoctonia solani AG1 IA Induces Cell Death and Triggers Defence Response in Nicotiana tabacum
Source: Mol Plant Pathol. 2025 Mar 17;26(3):e70075. doi: 10.1111/mpp.70075 (PMC11911542; doi:10.1111/mpp.70075)
Supplement: Supplementary file 9 — Figure S9. [file MPP-26-e70075-s001.docx]

**
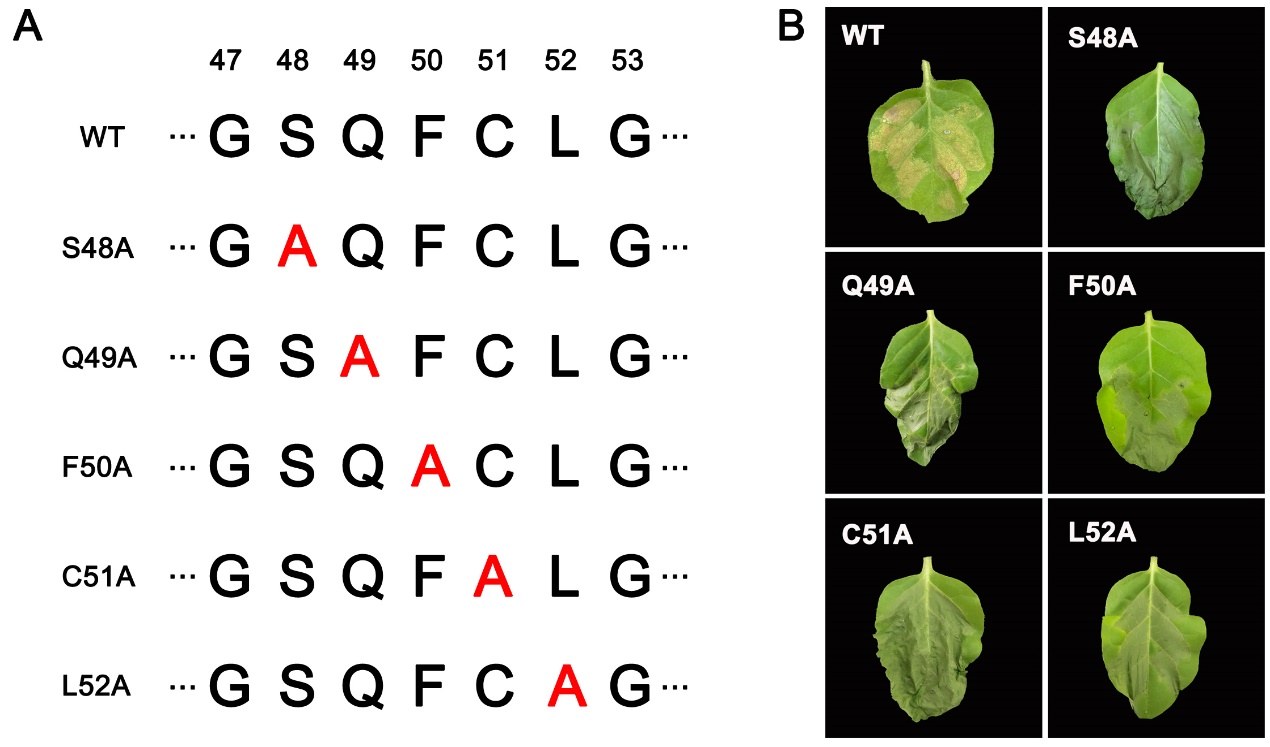
**

**Figure S9 Single amino acid substitution in RsEG146^C47-53^ induced cell death of *N. tabacum*. A,** Schematic presentation of the examined site-directed mutants. Each amino acid from 48 to 52 was substituted to alanine respectively. **B**, Analysis of amino acid residue inducing cell death by transiently expressing mutants in *N. tabacum* leaves. All mutants were maintained necrosis activity.
