# Supplementary material for: Glycoside Hydrolase Family 16 Enzyme RsEG146 From Rhizoctonia solani AG1 IA Induces Cell Death and Triggers Defence Response in Nicotiana tabacum
Source: Mol Plant Pathol. 2025 Mar 17;26(3):e70075. doi: 10.1111/mpp.70075 (PMC11911542; doi:10.1111/mpp.70075)
Supplement: Supplementary file 10 — Figure S10. [file MPP-26-e70075-s012.docx]

**
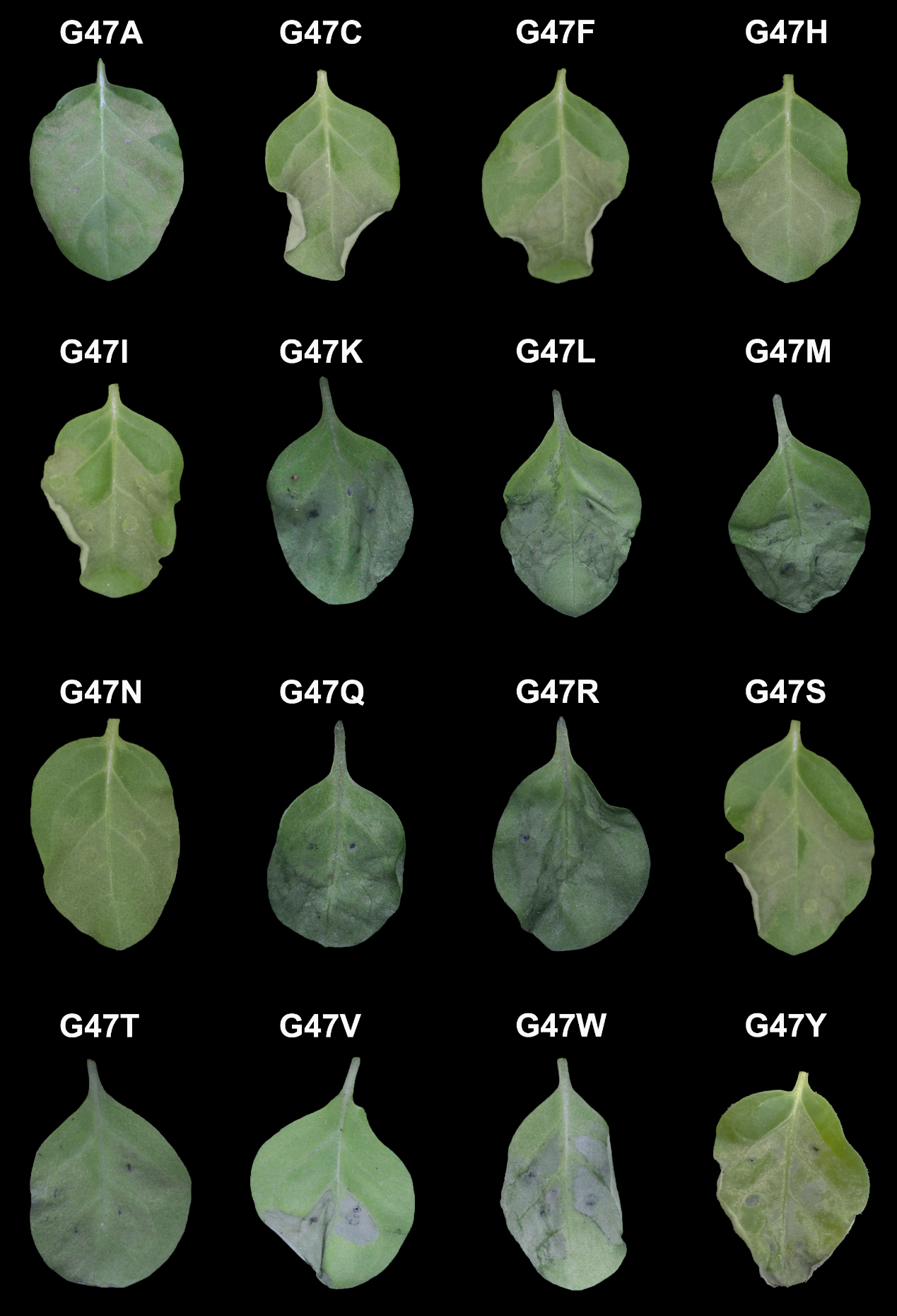
**

**Figure S10 Saturation mutagenesis of Gly47 in RsEG146^C23-70^ induced cell death in *N. tabacum* leaves except mutant G47D, G47E and G47P.**
