# Supplementary material for: Glycoside Hydrolase Family 16 Enzyme RsEG146 From Rhizoctonia solani AG1 IA Induces Cell Death and Triggers Defence Response in Nicotiana tabacum
Source: Mol Plant Pathol. 2025 Mar 17;26(3):e70075. doi: 10.1111/mpp.70075 (PMC11911542; doi:10.1111/mpp.70075)
Supplement: Supplementary file 11 — Figure S11. [file MPP-26-e70075-s009.docx]

**
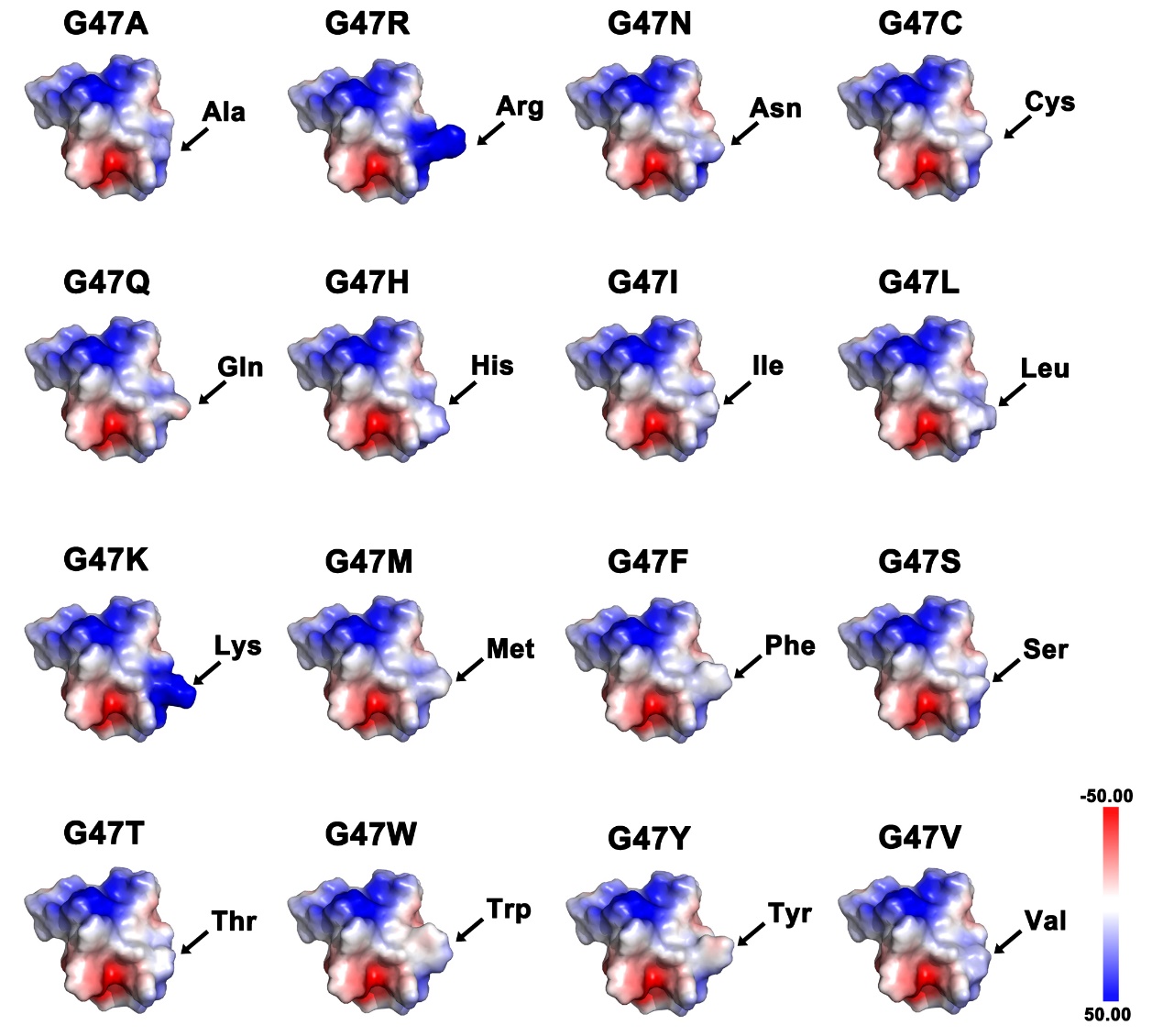
**

**kcal·mol^-1^**

**Figure S11 Protein structures and electrostatics potential analysis of Gly47 mutants of RsEG146 ChtBD1 domain except G47D, G47E and G47P.**
