# Supplementary material for: Glycoside Hydrolase Family 16 Enzyme RsEG146 From Rhizoctonia solani AG1 IA Induces Cell Death and Triggers Defence Response in Nicotiana tabacum
Source: Mol Plant Pathol. 2025 Mar 17;26(3):e70075. doi: 10.1111/mpp.70075 (PMC11911542; doi:10.1111/mpp.70075)
Supplement: Supplementary file 12 — Figure S12. [file MPP-26-e70075-s011.docx]

**
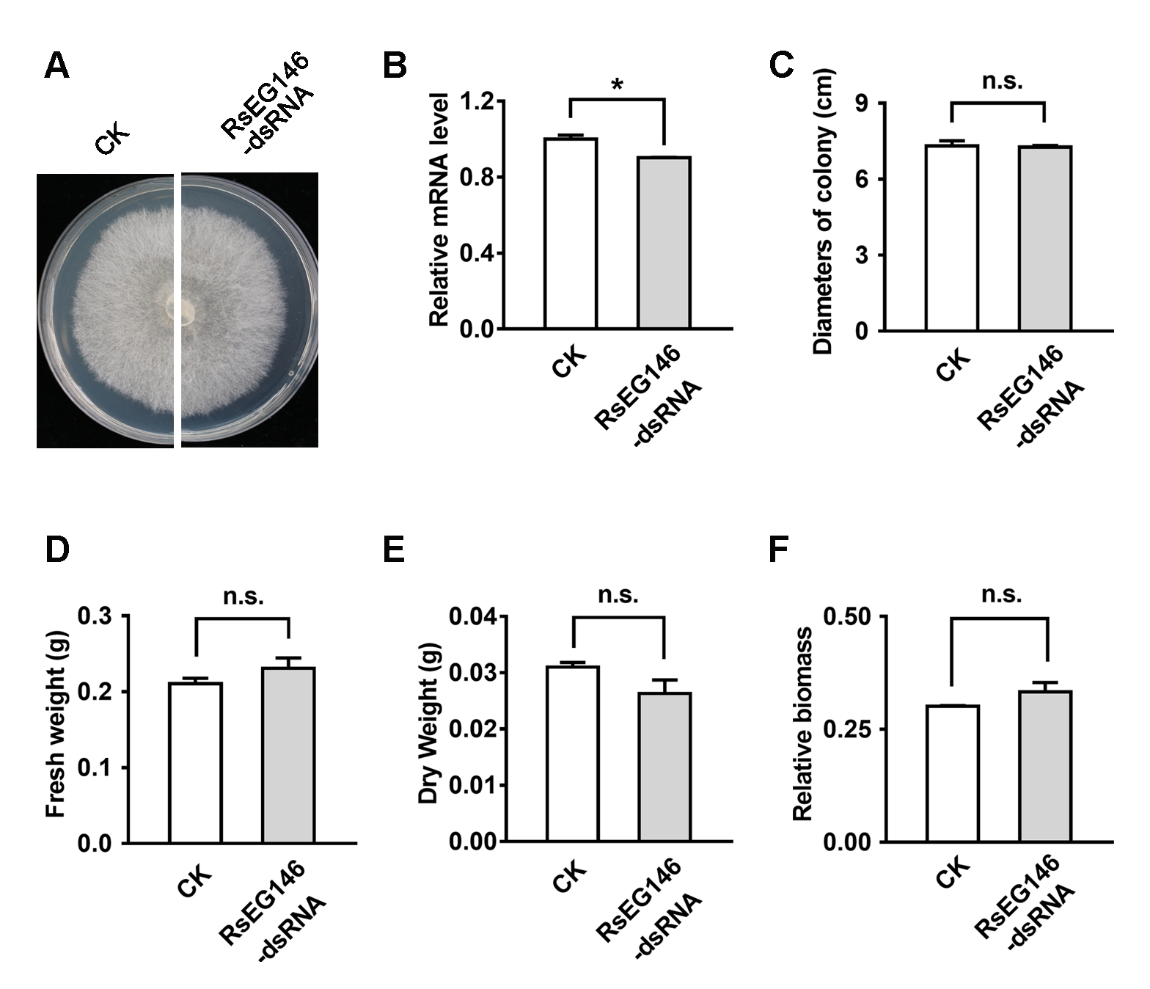
**

**Figure S12 RsEG146 silenced by spray-induced gene silencing does not affect mycelial growth of *R. solani*. A-E,** Colonial morphology, fresh weight and dry weight of mycelia and RsEG146 expression level of *R. solani* after treating by RsEG146-dsRNA. **F,** Biomass of *R. solani* in diseased *Z. mays* leaves. Column = mean ± SE (*P < 0.05, **P<0.01).
